# p63 Directs Subtype-Specific Gene Expression in HPV+ Head and Neck Squamous Cell Carcinoma

**DOI:** 10.3389/fonc.2022.879054

**Published:** 2022-05-31

**Authors:** Alexandra Ruth Glathar, Akinsola Oyelakin, Christian Gluck, Jonathan Bard, Satrajit Sinha

**Affiliations:** Jacobs School of Medicine and Biomedical Sciences, Department of Biochemistry, University at Buffalo, Buffalo, NY, United States

**Keywords:** p63, HPV, HNSCC, gene expression profiling, biomarker

## Abstract

The complex heterogeneity of head and neck squamous cell carcinoma (HNSCC) reflects a diverse underlying etiology. This heterogeneity is also apparent within Human Papillomavirus-positive (HPV+) HNSCC subtypes, which have distinct gene expression profiles and patient outcomes. One aggressive HPV+ HNSCC subtype is characterized by elevated expression of genes involved in keratinization, a process regulated by the oncogenic transcription factor ΔNp63. Furthermore, the human *TP63* gene locus is a frequent HPV integration site and HPV oncoproteins drive ΔNp63 expression, suggesting an unexplored functional link between ΔNp63 and HPV+ HNSCC. Here we show that HPV+ HNSCCs can be molecularly stratified according to ΔNp63 expression levels and derive a ΔNp63-associated gene signature profile for such tumors. We leveraged RNA-seq data from p63 knockdown cells and ChIP-seq data for p63 and histone marks from two ΔNp63^high^ HPV+ HNSCC cell lines to identify an epigenetically refined ΔNp63 cistrome. Our integrated analyses reveal crucial ΔNp63-bound super-enhancers likely to mediate HPV+ HNSCC subtype-specific gene expression that is anchored, in part, by the PI3K-mTOR pathway. These findings implicate ΔNp63 as a key regulator of essential oncogenic pathways in a subtype of HPV+ HNSCC that can be exploited as a biomarker for patient stratification and treatment choices.

## Introduction

Head and neck squamous cell carcinoma (HNSCC) is the sixth most common cancer worldwide and has a 5 year-mortality rate of nearly 50%, making it a leading cause of cancer-related death (1). HPV infection has overtaken alcohol and tobacco consumption as the predominant risk factor in the majority of newly diagnosed HNSCC cases (2–4). Intriguingly, patients with HPV+ HNSCC have better overall survival and progression-free survival than those with HPV− HNSCC. However, current treatment options for both HPV+ and HPV− HNSCCs consist of standard care regimens of chemoradiotherapy concurrent with cisplatin (2, 5). More attention has been given toward de-escalation of therapy for HPV+ HNSCC, and a clearer understanding of the underlying biology may aid in identifying patients who would benefit from new treatment modalities (6).

HPV infection of epithelial cells, primarily in the oropharynx, can result in the integration of the viral genome into the host genome, leading to dysregulated expression of viral and cellular oncoproteins and carcinogenesis (7). HPV E6 and E7 oncoproteins are the primary drivers of the pathogenesis of HPV and function by degrading tumor suppressor p53 and retinoblastoma protein (pRb), respectively, leading to activation of the cell cycle-promoting E2F family of transcription factors (TFs) (8, 9). Integration of the HPV genome alters the expression and DNA methylation profiles of a broad range of host genes (10). Although the HPV genome can integrate throughout the human genome, it occurs with a higher incidence in some regions, including the 3q region surrounding *TP63* (11–14). *TP63* encodes p63, a member of the p53 family of transcription factors, which plays an essential role in the development and maintenance of the stratified squamous epithelium (15–18). ΔNp63α is the most prevalent p63 isoform in tissues of epithelial origin and acts predominantly as an oncogene in several cancers, including HNSCC, while TAp63 has much more restricted expression and shows tumor-suppressor features (18–22). Ectopic expression of HPV oncoproteins in human keratinocytes leads to upregulation of p63 at both the mRNA and protein levels (23). Conversely, silencing of E6/E7 expression in HPV+ cell lines leads to a loss of p63 expression (23). Despite these known functional interactions between p63 and HPV, very few studies have examined the specific role of p63 in modulating gene expression in HPV+ HNSCC.

Hierarchical clustering analyses of HPV+ HNSCCs revealed two distinct subtypes based on gene expression profiles, copy number alterations, mutational profiles, and patient outcomes (11, 14). One subtype characterized by the amplification of the 3q chromosomal region, including the *TP63* locus, was shown by two independent studies to be enriched in pathways involved in keratinization and cell adhesion (11, 14). Interestingly, patients with this subtype tend to have worse outcomes and respond more poorly to treatment compared to patients with tumors belonging to the other HPV+ subtype (11, 14). These studies suggest that there are HPV-dependent mechanisms that affect p63 function in HPV+ HNSCC and the pathology of this disease.

To explore the oncogenic role of p63 in HPV+ HNSCC, we established a p63-driven gene regulatory network based on both preclinical cell culture models and tumor datasets. Our in-depth examination of p63 in the broader transcriptomic and genomic context revealed that p63 regulates critical sets of genes and pathways in the HPV infection pathway and HPV-associated malignancy, including PI3K signaling, WNT signaling, and cell cycle control which may inform the clinical differences between the HPV+ HNSCC subtypes. Importantly, we found that p63 expression correlates with the more aggressive HPV+ HNSCC subtype and that it directs the associated gene expression programs. Finally, we identified a potentially important role for p63 in regulating PI3K signaling and mTOR signaling in HPV+ HNSCC, which may have implications for future treatment choices. Our studies suggest that p63 is an important driver of the subtype-specific gene expression program in HPV+ HNSCC, and can serve as a biomarker to identify patients with more aggressive disease.

## Material and Methods

### Cell Culture Studies

The UM-SCC-104 (referred to as SCC104) cell line was obtained from Sigma-Aldrich, and the UPCI : SCC152 cell line (referred to as SCC152) was obtained from ATCC. Both SCC104 and SCC152 cell lines have been reported to be HPV-16 positive (24, 25). SCC25 and SCC47 cell lines were purchased from ATCC and Millipore Sigma, respectively. Cell lines UM-SCC-11B, UM-SCC-74A, UM-SCC-29, UM-SCC-23, and UM-SCC-103 were obtained from Dr. Thomas Carey (University of Michigan). HSC-3 and CAL-27 cell lines were generously provided by Dr. Manish Bais (Boston University). All cell lines were grown and maintained in high-glutamine DMEM or DMED/F12 as recommended, with the following supplements: 10% FBS, 1% nonessential amino acids, and antibiotics. Other cell lines used in this study have been described before in Gluck et al. 2019 (26). The identities of the cell lines utilized in this study were confirmed *via* short tandem repeat profiling through services offered by Genetica. All cell lines were tested by the eMycoPlus Mycoplasma PCR Detection Kit (BulldogBio) to ensure that they were bereft of any mycoplasma infection.

### Knockdown of p63

Lentivirus-mediated depletion of p63 in SCC104 and SCC152 cells was performed using the pGIPZ system. GIPZ lentiviral shRNAs (clone IDs V2LHS_24248 [sh1] and V2LHS_24250 [sh2]) targeting *TP63* were obtained from and virus was generated with the help of Gene Modulation Services Shared Core at Roswell Park Comprehensive Cancer Center. Viral infection and selection with Puromycin was performed as described before (27).

### Western Blot Analysis

Protein extracts were prepared according to previously published protocol (27). Briefly 5 μL of protein lysates were loaded onto SDS-polyacrylamide gels and transferred to Immun-Blot PVDF membranes (Bio-Rad Laboratories). After blocking in 5% milk, the membranes were incubated in primary antibodies against the following: p63 (4A4, 1:20,000), ΔNp63 (E6Q3O; Cell Signaling Technology, 1:5000), ITGB1 (Proteintech, 1:10,000), ITGB4 (Proteintech, 1:10,000), cMYC (Santa Cruz Biotechnology, 1:5000), AKT1 (Proteintech, 1:10,000), mTOR (Proteintech, 1:10,000), Raptor (Proteintech, 1:10,000), S6 (Cell Signaling Technology, 1:5000), and p-S6 (Cell Signaling Technology, 1:5000). The MAB374 antibody (EMD Millipore) was used to detect GAPDH as a loading control at 1:20,000 dilution. HRP-conjugated secondary antibodies corresponding to the primary antibody host were incubated with each blot. Unbound antibodies were washed off in 0.05% Tween-20 in Tris-buffered saline. The LumiGLO peroxidase chemiluminescent substrate kit (SeraCare) was used to detect antibody-labeled proteins, and membranes were imaged using the Bio-Rad ChemiDoc imaging system.

### ChIP of p63 and Histone Marks

The iDeal ChIP-seq kit for transcription factors (C01010055; Diagenode) or for histones (C01010051; Diagenode) and the associated protocols were used to perform ChIP-seq. SCC104 and SCC152 cells were grown to ~90% confluency and cross-linked in the supplied fixation buffer supplemented with 0.5% formaldehyde for 10 min. Lysates from the fixed cells were subsequently sonicated with a Diagenode Bioruptor to obtain sheared chromatin with an approximate fragment length of 150–400 bp. The ChIPs for p63 were carried out using 2 μg of p63 4A4 antibody (Santa Cruz Biotechnology) and 2 μg of ΔNp63-1.1 antibody (28). After cross-link reversal, proteinase-K/RNase A treatment, and DNA purification, libraries were prepared using the ThruPLEX DNA-seq kit (Rubicon Genomics). ChIP DNA and input controls were then subjected to 50-bp single-end sequencing on an Illumina HiSeq 2500, which resulted in 15–25 million reads per sample.

### ChIP-Seq Analysis

The raw ChIP-seq reads from all experiments were mapped to the *Homo sapiens* genome (hg19 build) using Bowtie v1.1.1 with the parameter m=1 to remove all reads mapping to multiple genomic loci (29). Peak calling was then performed using MACS2 v2.1.0 with a minimum FDR cutoff of 0.05 and sequenced Input used as control for each experiment, and resultant peaks were matched to the nearest gene using GREAT analysis with default settings (30, 31). For visualization of ChIP peaks, the package deeptools v3.3.2 was used to preprocess bam files to generate bigwig files which were then uploaded to IGV (32). Adobe illustrator was used for final image processing. Peak summits determined by MACS2 v2.1.0 were used as input to HOMER’s findMotifsGenome.pl program with the parameter “-size 200” (33).

### RNA Isolation and Library Preparation for RNA-Seq

Total RNA from cell lines was extracted using a Direct-zol RNA miniprep kit (Zymo Research). The extracted RNA was snap-frozen on dry ice and stored at −80°CC until library preparation. For each RNA sample, cDNA libraries were prepared using the TrueSeq RNA sample preparation kit (Illumina) and were then 50-bp single-end sequenced or paired-end sequenced on an Illumina HiSeq 2500. Quality control metrics were performed on raw sequencing reads using the FASTQC v0.11.9 application.

### RNA-Seq Analysis

Reads were mapped to the appropriate reference genome, GRCh38/hg19 build, with HISAT2 v2.1.0 (34). Reads aligning to the reference genome were quantified with featureCounts v1.5.3 to generate a matrix of raw counts, which was then processed in R, to generate normalized expression values in transcripts per million according to the method proposed by Wagner et al. (35). Differential gene expression analysis comparing control to p63 knockdown was carried out using DESeq2 v1.24.0 (26). DEGS with an FDR value of ≤ 0.1 were considered statistically significant.

### qRT-PCR Analysis

Total RNA from SCC104 and SCC152 knockdown cell lines was extracted using a Direct-zol RNA miniprep kit (Zymo Research). RNA was reverse transcribed with the Bio-Rad iScript cDNA synthesis kit according to the manufacturer’s instructions. The resulting cDNA was used for qPCR with Bio-Rad iQ SYBR green Supermix. A list of the qRT-PCR primers can be found in **Supplementary Table 7**.

### HNSCC Dataset Analysis

RNA-seq data from patient samples were obtained from GEO (GSE122512, GSE112026, GSE74927, and GSE72536) (11, 36–38). HPV+ tumors were assigned based on data presented in the original paper of each dataset. Alignment and quantification of counts for each dataset were performed as indicated by the original study. TCGA RNA-seq expression and CNA datasets were downloaded from cBioPortal (39, 40). Briefly, RNA-seq counts were extracted and normalized using the median-ratio method (DESeq2 v.1.24.0 [75]) and subsequently transformed to transcript per million values (35). For GSE112026, RSEM values were utilized for transcript quantification. HPV+ tumors were segregated into high and low p63 expression groups based on the median p63 expression level calculated from the RNA-seq data.

### Determination of Enhancers and SEs According to H3K27Ac Marks

H3K27Ac ChIP-seq data from SCC104 and SCC152 cells were aligned to the human genome as described above. Narrow peaks were called using MACS2 v2.1.0 using the following parameters: -p 0.01, -nomodel, -extsize 150. The resulting narrowPeaks files were converted to gff format and used as inputs for the ROSE (rank order of super-enhancers) algorithm, which was run using default parameters along with appropriate input controls to generate typical enhancer and SE lists (41, 42).

### Histone Modification Enrichment at p63 Binding Sites

The deepTools package was utilized to generate a signal matrix of histone modifications H3K27Ac, H3K4Me1, and H3K4Me3. The fluff python package was then utilized to generate heat maps showing the resulting signal of the histone modifications around a 2-kb window centered at each p63 ChIP-seq peak summit. The resulting histone signal enrichment was subjected to k-means clustering (k=3) (43).

### Genomic Feature Assignment

The CEAS tool was used to annotate p63 ChIP-seq peaks to the nearest genomic feature of the hg19 genome assembly (44). The promoter region was considered up to 1,000 bp from a transcriptional start site, and the proximal enhancer was considered from 1,000 to 3,000 bp away. Any binding within a gene was considered intragenic, whereas any binding site greater than 3,000 bp upstream or downstream was considered distal intergenic.

### Motif Enrichment Analysis of Enhancers

To determine the top enriched DNA binding motifs of TFs found within nucleosome-free regions of SCC104 and SCC152 SEs, nucleosome-free regions were first determined using the HOMER findPeaks tool with the -nfr flag (33). The AME tool was used to determine enriched motifs found within the HOCOMOCO Human (v11 CORE) database. Motifs were ranked according to *p* value.

### Gene Ontology/Pathway Enrichment Analysis

The GREAT tool was used to annotate binding loci to the nearest gene (31). Identified genes were then subjected to KEGG pathway analysis utilizing the DAVID functional annotation tool (45–47). For RNA-seq data, DEGs were subjected to both KEGG analysis utilizing the DAVID functional annotation tool and canonical pathway analysis by gene set enrichment analysis (48).

### Statistics

Statistical analyses were performed using R, a free software environment for statistical computing and graphics. A Shapiro-Wilk test was performed to check the normality of data, and then either a student’s *t* test or Wilcoxon signed-rank test was performed according to whether the data were normally distributed. A *p* value lower than 0.05 was considered significant.

## Results

### Generation of a p63 Gene Signature From the HPV+ HNSCC TCGA Tumor Dataset

Although a broad oncogenic role of p63 in HNSCC has been reported (21, 49, 50), its specific role in the HPV+ subtypes has not been fully explored. Thus, we first examined three independent RNA-seq datasets of HPV+ HNSCC tumors (GEO datasets GSE112026, GSE74927, and GSE72536) and observed a gradient in the pattern of p63 mRNA expression (**Supplementary Figures 1A–C**). Segregation of HPV+ HNSCC tumors according to median p63 expression revealed distinct p63^high^ and p63^low^ subtypes. This distinction was in agreement with previous unsupervised gene expression clustering analyses performed on HPV+ HNSCC tumors that had identified subtypes with distinct gene expression patterns, including different p63 levels (11, 14, 51).

**Figure 1:**
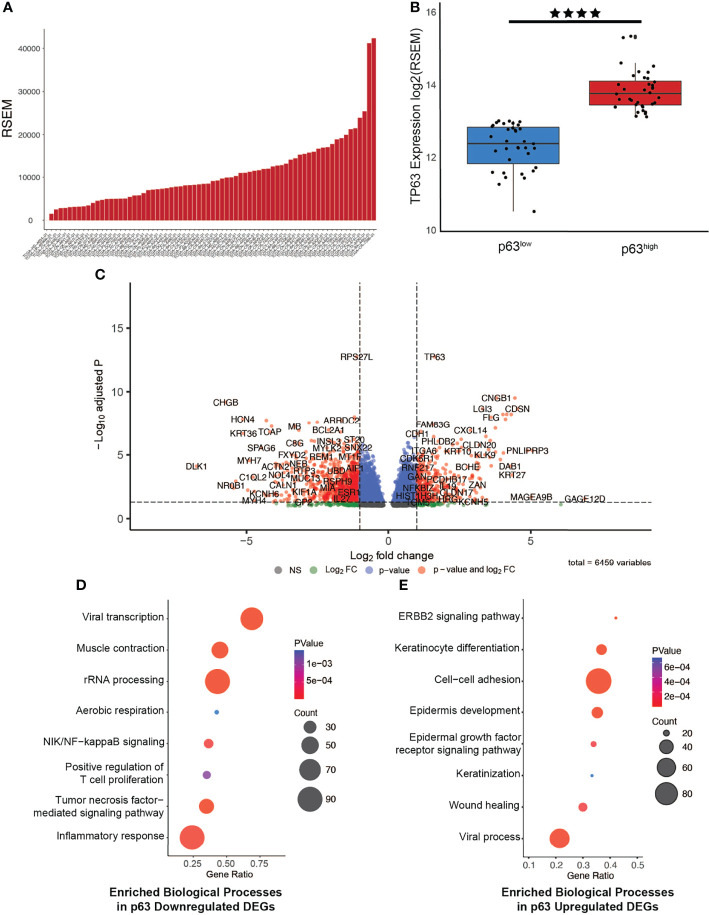
Generation of a p63-driven gene signature in HPV+ HNSCC TCGA tumors. **(A)** Bar chart of p63 expression across tumors in the TCGA HPV+ HNSCC dataset. **(B)** Boxplots displaying the significant difference in expression of *TP63* between the groups (*p* value = 2.472e-6). Tumors were segregated into p63^low^ and p63^high^ groups on the basis of median p63 expression. **(C)** Volcano plot of identified DEGs between p63^low^ and p63^high^ samples. *TP63* is shown as one of the most significant DEGs. **(D)** Gene Ontology (GO) based biological pathway analysis of DEGs whose expression was downregulated according to p63 expression. **(E)** GO biological pathway analysis of DEGs whose expression was upregulated according to p63 expression. **** symbol means a *p*-value of ≤ 0.0001.

To investigate the functional relevance of p63 in HPV+ HNSCC, we next focused on data from 67 HPV+ tumors that are available in The Cancer Genome Atlas (TCGA) patient datasets. Exploration of p63 expression across the HPV+ HNSCC tumors in this dataset revealed a similar pattern of p63 expression as observed in the GEO dataset (**Figure 1A**). Using the RNA-seq data from the TCGA datasets to segregate the HPV+ tumors according to p63^high^ and p63^low^ expression, we identified 6,459 differentially expressed genes (DEGs) between these two populations (**Figures 1B, C** and **Supplementary Table 1**). We next examined GO biological processes that were enriched with upregulated and downregulated DEGs to identify pathways that are likely influenced by p63. Downregulated DEGs were significantly enriched in pathways involved in viral transcription and inflammatory immune responses, such as NF-κB and tumor necrosis factor signaling (**Figure 1D**). Upregulated DEGs were significantly enriched in pathways associated with cell adhesion and keratinization—processes linked to p63 (**Figure 1E**). These findings were of particular interest in lieu of prior HPV+ subtype studies in which gene expression-driven clustering analysis showed differential enrichment of immune response and cell adhesion pathways. Notably, the clustering of tumors according to p63 expression recapitulated the molecularly defined distinct subtypes of HPV+ HNSCC.

### Mapping the Genomic Targets of p63 in Representative HPV+ HNSCC Cell Lines

To verify that the pattern of p63 expression in tumors matches that in HNSCC cell lines to serve as suitable models for follow-up studies, we examined RNA-seq data generated from 9 HPV+ and 55 HPV− HNSCC cell lines (**Supplementary Figure 2A**) (38). Several of the HPV+ HNSCC cell lines had high p63 expression, and we verified this expression at the protein level by Western blotting with two anti-p63 antibodies. Similar to previous reports (23), four of the five HPV+ HNSCC cell lines consistently showed high levels of p63 protein expression, specifically the ΔNp63 isoform (**Supplementary Figure 2B**). Of these, we chose the SCC104 and SCC152 cell lines for follow-up mechanistic studies. These two well-characterized cell lines have been confirmed for HPV-positivity and shown to express viral factors and oncogenes E6 and E7, making them suitable for studies of HPV+ HNSCC *in vitro* (25). The SCC104 cell line was the primary choice for most of our experiments because of its robust growth and detailed phenotypic characterization compared to that for SCC152 (24); data from the SCC152 cell line were used to corroborate and/or validate the findings.

**Figure 2:**
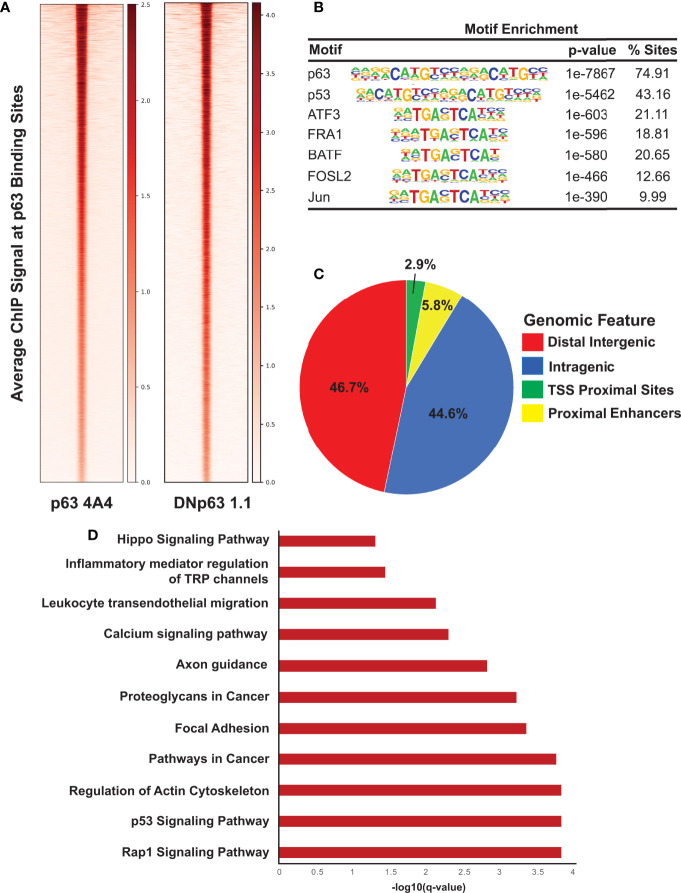
ChIP-seq analysis reveals direct p63 targets in SCC104 cells. **(A)** Heatmap of the average ChIP-seq signals from p63 binding sites for two p63 antibodies across the genome. **(B)** Top transcription factor motifs derived from HOMER’s motif analysis on SCC104 consensus p63 ChIP peaks. **(C)** Distribution pattern of genomic features associated with p63 binding sites across the genome. **(D)** Bar graphs displaying selected top enriched KEGG pathways associated with genes identified through GREAT analysis of the top 2,500 p63 ChIP-seq peaks.

To identify the global network of p63 target genes, we performed ChIP-seq experiments in SCC104 cells with two anti-p63 antibodies. ChIP-seq of p63 with the widely used 4A4 antibody that recognizes all p63 isoforms identified 18,085 genomic sites, whereas ChIP-seq with a ΔNp63-specific antibody, ΔNp63-1.1, identified 10,028 p63-bound sites (**Figure 2A**); 9,724 sites were identified by both antibodies, which were deemed high-confidence p63 targets and utilized for subsequent analysis (**Supplementary Table 2**). As expected, analysis of the p63 ChIP-seq peaks using HOMER revealed the consensus p63 motif (*p* = 1e-7867) as the most highly enriched motif, followed by the p53 motif (*p* = 1e-5462), which was independently confirmed by using MEME-ChIP (**Figure 2B** and **Supplementary Figure 3B**). Other enriched motifs were for TFs belonging to the AP-1 family, which cooperates with p63 to regulate target gene expression (**Figure 2B**) (52). The distribution of p63 peaks relative to the transcriptional start sites revealed that, in both cell lines, p63 preferentially targets intragenic and distal regulatory regions, which are likely to act as enhancer sites (**Figure 2C** and **Supplementary Figure 3C**). A DAVID-based pathway analysis of the genes associated with the top 2,500 p63 ChIP-seq peaks revealed several important pathways, including those deemed important in HPV-associated cancers, such as focal adhesion, p53, and Rap1 signaling pathways (**Figure 2D**) (53, 54). Interestingly, focal adhesion pathways are enriched in the subtype of HPV+ HNSCC tumors with high p63 expression levels (11, 14, 51). In parallel, we performed ChIP-seq of p63 in SCC152 utilizing the 4A4 antibody and identified 26,255 genomic peaks, with the p63 motif as the most enriched (**Supplementary Figures 3A, B** and **Supplementary Table 2**). p63 binding-associated genes in SCC152 were enriched in pathways involving MAPK signaling, cell adhesion molecules, and Rap1, which all play a role in HPV infection (**Supplementary Figure 3D**). Of the 9,724 high-confidence p63 binding sites (identified with both antibodies), 5,933 sites were shared between SCC104 and SCC152 cell lines, providing a strong list of bona-fide p63 targets in HPV+ HNSCC.

### Characterizing the Enhancer Landscape of p63^high^ HPV+ Cells

We next explored the epigenomic landscape of SCC104 and SCC152 by ChIP-seq using histone marks H3K27Ac, H3K4Me1, and H3K4Me3 (**Supplementary Figures 4A**, **5A**) (55), which identify gene regulatory features such as active enhancers (H3K27Ac^high^ and H3K4Me1^high^), active promoters (H3K27Ac^high^ and H3K4Me3^high^), and poised enhancers (H3K27Ac^low^ and H3K4Me1^high^) (56).We performed k-means clustering on the histone marks centering around each H3K27Ac peak as described in Gluck et al. (27), which identified three distinct clusters of regulatory elements. In SCC104 cells, clusters 1 and 3 represented active enhancers, and associated genes were enriched in pathways such as mRNA processing, differentiation, and focal adhesion (**Supplementary Figures 4B**). Cluster 2 represented active promoters, and associated genes were enriched in viral processes and cell motility (**Supplementary Figures 4A, B**). To identify which TFs may regulate enhancer expression in these clusters, we performed a motif analysis and found enrichment of ZNF, IRF, KLF, and Ets family motifs as well as E2F motifs (**Supplementary Figure 4C**).

Similar results were obtained in clustering analysis of SCC152 cells, where clusters 1 and 3 also represented active enhancers, and genes associated with these sites were enriched in focal adhesion, cell junction assembly, and Notch signaling pathways (**Supplementary Figure 5B**). Cluster 2 was similarly associated with active promoters, and associated genes were enriched in mRNA processing and cell-cell adhesion pathways (**Supplementary Figures 5A, B**). Motif analysis of these regions in SCC152 also showed enrichment of E2F motifs within all identified clusters (**Supplementary Figure 5C**). The enrichment of E2F motifs across various gene regulatory elements in both HPV+ HNSCC cell lines is interesting and likely to be relevant given the known interaction of E2F TFs and HPV E7 and its effects on downstream pathways in HPV+ disease.

### p63 Is Super-Enhancer Marked and Regulates Expression of Super-Enhancer-Associated Genes in HPV+ HNSCC

**Figure 3:**
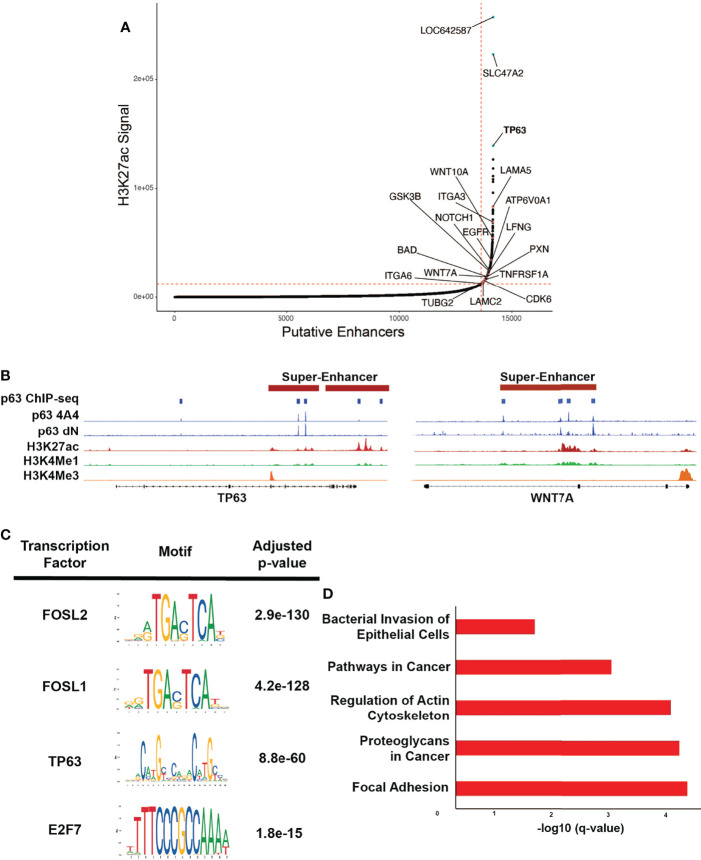
p63 binding enriched at SEs in SCC104. **(A)** Hockey plot displaying the ranked H3K27Ac ChIP-seq signal in SCC104 cells. Representative genes marked by super-enhancers (SE) are shown. *TP63* is highlighted in blue as one of the top SE-associated genes in SCC104. Other labeled points represent genes that have been found in previous literature to be associated with HPV infection. **(B)** Integrative Genomics Viewer (IGV) based representation of Histone and p63 ChIP-seq data from SCC104 cells showcasing peaks of binding around the *TP63* and *WNT7A* loci. **(C)** Top enriched transcription factor motifs found in SE regions in SCC104 cells. **(D)** Bar graphs displaying selected top enriched KEGG pathways associated with genes identified through ROSE analysis of the SE landscape in SCC104 cells.

Multi-cluster enhancers, often referred to as super-enhancers (SEs), are associated with H3K27Ac^high^ marks and are densely occupied by key TFs (42, 57). These SEs are often associated with cell identity and lineage-driving genes and oncogenes, and in the context of HPV, viral oncoproteins have been found to play a central role in their activation (58, 59). It is postulated that SEs at the site of HPV integration likely upregulate the expression of HPV E6/E7, leading to activation of other SEs that facilitate disease progression (60). By applying the ROSE algorithm to the H3K27Ac ChIP-seq data, we identified 528 SEs in SCC104 and 317 SEs in SCC152 (**Figure 3A** and **Supplementary Figure 6A**, **Supplementary Table 3**) (42, 61). As expected, several SEs were associated with master TFs, including p63, but we also observed SEs associated with genes important to HPV infection and HPV-induced carcinogenesis, such as *WNT7A*, *ITGA2*, and *NOTCH1*. (**Figure 3B** and **Supplementary Figure 6B**; **Supplementary Table 3**) (62, 63). Motifs for TFs with known roles in HNSCC, including FOSL1, E2F1, and E2F7 (10, 64), were also enriched at SCC104 SEs (**Figure 3C** and **Supplementary Figure 6C**). We found significant enrichment of the p63 motif in SCC104 SEs, suggesting p63 regulates the transcription of many SE-associated genes in HPV+ HNSCC (**Figure 3C** and **Supplementary Figure 6C**). The enrichment of the p63 motif in SEs is functionally relevant, because most (369/540) were occupied by p63 according to the ChIP-seq results from SCC104 cells.

Analysis of SE-marked genes revealed notable enrichment of pathways associated with cancer, including those for focal adhesion and those involving proteoglycans (**Figure 3D**). Interestingly, we found that *SDC1*, which encodes protein syndecan-1 and is a direct p63 target, was associated with SEs (**Supplementary Table 3**). This is notable because syndecan-1 is the most abundant heparan sulfate proteoglycan in keratinocytes and serves as the primary HPV attachment receptor (65). HPV infection is known to affect the expression of genes involved with cell adhesion and cell motility, and several of the implicated genes were found in our data, including *LAMA5*, *ITGA3*, *ITGA6*, and *LAMC2* (11, 36, 54, 66). SE-marked genes in SCC152 were similarly enriched in pathways important for cancer, including the Hippo signaling pathway, implicated in HPV-associated oropharyngeal SCC (**Supplementary Figure 6D**) (67).

We next explored the epigenomic state (as defined by histone marks) of the gene regulatory regions bound by p63 that were identified by ChIP-seq. For this purpose, we performed k-means clustering of the three histone marks, which again identified three distinct clusters (**Supplementary Figures 7A**, **8A**). In SCC104 cells, cluster 1 represented active promoters, and genes associated with these sites were enriched in pathways for apoptosis, cell adhesion, and motility (**Supplementary Figure 7B**). Clusters 2 and 3 represented active enhancers, and the corresponding genes were associated with Notch, protein kinase B, and Rho signaling (**Supplementary Figures 7A, B**). Unsurprisingly, we observed enrichment of AP-1 motifs in p63-bound enhancer regions regulating such processes as signaling and differentiation, which has been reported in keratinocytes and breast cancer (68). Interestingly, we observed enrichment of E2F motifs in cluster 1, suggesting the interaction between p63 and E2F at active promoter regions regulates cellular movement and apoptosis (**Supplementary Figure 7C**).

Similar analyses in SCC152 cells showed identical patterns of clustering of gene regulatory elements, with cluster 1 representing active promoters and clusters 2 and 3 representing active enhancers (**Supplementary Figure 8A**). Cluster 1 showed enrichment of pathways associated with apoptosis and cellular organization (**Supplementary Figure 8B**). Unlike that in SCC104 cells, we did not find enrichment of E2F motifs in these regions but instead saw enrichment of FOX motifs (**Supplementary Figure 8C**). Genes found in clusters 2 and 3 were associated with tissue development, Notch signaling, and cell adhesion pathways (**Supplementary Figure 8B**). Similar to that for SCC104 cells, there was enrichment of ZNF, IRF, and ETS family motifs; however, there was no enrichment of AP-1 motifs (**Supplementary Figure 8C**). These findings suggest that p63 actively regulates genes and pathways considered important in HPV-induced carcinogenesis and that p63 may interact with cellular E2Fs at gene regulatory regions.

### Loss of p63 Expression Dysregulates Signaling Pathways Involved in HPV-Associated Carcinogenesis

**Figure 4:**
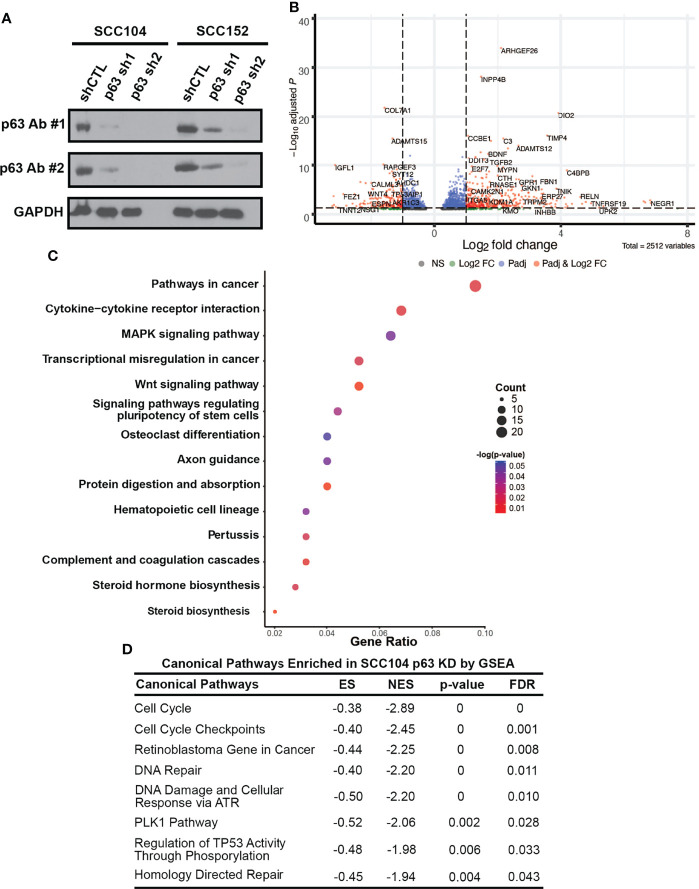
p63 knockdown in SCC104 shows enrichment of HPV-associated signaling pathways. **(A)** Western blot analysis of p63 expression in SCC104 and SCC152 cells expressing either p63-targeting shRNAs or a nontargeting shRNA (shCTL). GAPDH: loading control. **(B)** Volcano plot of DEGs resulting from p63 knockdown in SCC104 cells. **(C)** KEGG pathway analysis of SCC104 DEGs whose expression showed ≥log2 fold change of 1. **(D)** Gene set enrichment analysis (GSEA) of DEGs from p63 knockdown in SCC104 cells.

To identify p63 targets, we performed RNA-seq to profile the global transcriptomic changes resulting from loss of p63 expression. For these experiments, we stably knocked down p63 in SCC104 and SCC152 cells with two independent lentiviral mediated shRNAs. Western blotting confirmed that both shRNAs significantly reduced p63 expression, with sh2 showing markedly greater knockdown (**Figure 4A**). Loss of p63 substantially altered the transcriptomic landscape, with 6,607 and 5,809 genes exhibiting statistically meaningful changes in expression in SCC104 and SCC152 cells, respectively (**Supplementary Table 4**). Of these, 2,189 and 1,461 DEGs showed statistically meaningful (*p*_adj_ < 0.1) changes with both p63 shRNAs in SCC104 and SCC152 cells, respectively (**Figure 4B** and **Supplementary Figure 9A**; **Supplementary Table 4**).

To explore pathways affected by loss of p63, we focused on a select group of 615 DEGs (≥log2 fold change of 1) common to both shRNA knockdowns in SCC104 cells. KEGG analysis of these DEGs showed enrichment of many pathways important in HPV-associated carcinogenesis, such as WNT, MAPK, and PI3K-Akt signaling (**Figure 4C** and **Supplementary Figure 9B**) (21, 53, 62, 69, 70). We also performed gene set enrichment analysis of canonical pathways associated with these DEGs, which identified cell cycle and retinoblastoma gene in cancer categories (**Figure 4D**). Genes involved in the pRb signaling pathway, including *E2F1* and *CCNA2*, were significantly upregulated by the loss of p63, suggesting that p63 suppresses E2F-induced transcription and cell cycle activation.

The enrichment of pathways associated with HPV after p63 knockdown prompted us to look closer into the known HPV infection pathway. Of the 324 genes in the KEGG “human papillomavirus infection” pathway, 62 were differentially expressed upon p63 knockdown (**Supplementary Figure 10A**). HPV infection affects cell cycle regulation, which was also the case with p63 knockdown. Several factors involved in the cell cycle, including E2F1, RBL1, and cyclin A2, were upregulated upon loss of p63 (**Supplementary Figure 10A**). We also observed high enrichment of genes involved in focal adhesion and WNT and PI3K signaling (**Supplementary Figure 10B**).

### Generation of an Overall p63-Driven Gene Signature in HPV+ HNSCC

To delineate our high-stringency p63-driven gene signature, we combined the gene signatures we identified from TCGA tumor data and from the p63 expression-defined cell lines (**Supplementary Figure 11A** and **Supplementary Table 1**). To identify which genes are most reliant on p63 expression, we utilized our sh2 data from both cell lines to generate our HPV+ cell line-based signatures (**Supplementary Figure 11A** and **Supplementary Table 5**). These analyses identified 1,052 genes shared between the TCGA and SCC104 datasets and 827 genes shared between the TCGA and SCC152 datasets (**Supplementary Figure 11B**). Then, to identify genes which were directly regulated by p63, we incorporated our p63 ChIP-seq data, which revealed 498 and 574 genes that were directly bound by p63 in SCC104 and SCC152 cells, respectively (**Supplementary Figure 11B**). We filtered all genes that were common between analyses to generate our combined p63 signature of 420 genes (**Supplementary Table 5** and **Supplementary Figure 11B**). Finally, to identify genes of potential importance, we merged this gene signature with our super-enhancer landscape, which revealed 55 super-enhancer-associated genes (**Supplementary Figure 11B** and **Supplementary Table 5**). These analyses provided a p63-driven gene expression signature in HPV+ HNSCC that is relevant in both cancer and HPV contexts for follow-up studies.

### p63 May Be a Key Player in Subtype-Specific HPV+ HNSCC Gene Expression

**Figure 5:**
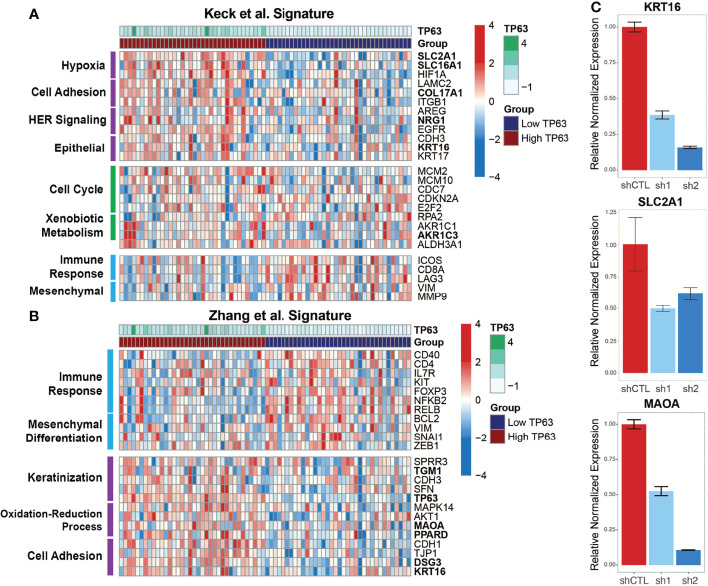
p63^high^ and p63^low^ groups recapitulate published HPV+ HNSCC subtype gene signatures. **(A)** Heatmap representation of expression of genes from the Keck et al. (14) signature within the p63^high^ and p63^low^ HPV+ HNSCC TCGA samples. DEGs are separated based on distinct patterns of expression between the p63^high^ and p63^low^ groups. Specific biological and signaling pathways associated with the DEGs are indicated. Bolded genes represent those that were included in the consensus p63-derived gene signature. **(B)** Heatmap representation of expression of genes from the Zhang et al. (11) signature within our p63^high^ and p63^low^ HPV+ HNSCC TCGA samples. DEGs are divided up by patterns of expression between the p63^high^ and p63^low^ groups. Specific biological and signaling pathways associated with the DEGs are indicated. Bolded genes represent those that were included in the consensus p63-derived gene signature. **(C)** Graph showing qRT-PCR results for normalized expression of 3 key genes in SCC104 cells expressing shCTL and p63 shRNAs.

To explore how p63 regulates subtype-specific gene expression in HPV+ HNSCC, we compared our combined p63-driven signature of 420 genes with the aforementioned subtype-specific signatures. Our previous analyses of transcriptomic changes upon p63 knockdown revealed enrichment of cell adhesion and keratinization pathways, like the HPV-KRT subtype defined by Keck et al. (14) and Zhang et al. (11). Keck et al. (14) found HPV-KRT tumors upregulate genes involved in hypoxia, cell adhesion, and HER signaling as well as epithelial-associated genes, whereas genes involved in immune response and mesenchymal-associated genes are downregulated. Our p63^high^ signature displayed a similar pattern of upregulated gene expression (compared to expression in p63^low^ samples), supporting the notion that high p63 expression is a defining aspect of the HPV-KRT subtype (**Figure 5A**). Interestingly, we also found that 36 of our 420 p63 signature genes, including *MAOA, SLC2A1, COL17A1*, and *KRT16*, were associated with the reported HPV-KRT subtype signature (**Supplementary Table 6**). Conversely, the p63^low^ expression signature was enriched with genes associated with the immune response and mesenchymal tissues (**Figure 5A**).

The HPV-KRT signature defined by Zhang et al. (11) had patterns of expression and pathway enrichment similar to the signature defined by Keck et al. (14). Accordingly, the gene signatures within our p63^high^ subgroups also showed comparable upregulation of genes associated with keratinization, redox processes, and cell adhesion previously seen by Zhang et al. (11) (relative to expression in the p63^low^ samples) (**Figure 5B**). The HPV-KRT-like subtype signature defined by Zhang et al. (11) was apparent in our p63-based DEGs (49 DEGs, including *TP63, MAOA, PPARD*, and *KRT16*) (**Figure 5B** and **Supplementary Table 6**), whereas the p63^low^ samples had upregulated genes associated with the immune response and mesenchymal differentiation (**Figure 5B**). To validate the differences in expression observed between the p63^high^ and p63^low^ groups and in our p63-driven signature, we performed qRT-PCR for several genes identified in each subtype signature, including *KRT16*, *SLC2A1*, and *MAOA*, in the HPV+ cell line with p63 knockdowns, confirming our RNA-seq findings (**Figure 5C**). Altogether, these findings support a role for p63 in subtype-specific gene expression within HPV+ HNSCC and suggest that p63 directs the specific gene expression profiles that were discovered by hierarchal clustering of global gene expression in HPV+ HNSCC.

### p63 Exerts Broad Control of the PI3K-Signaling Pathway

**Figure 6:**
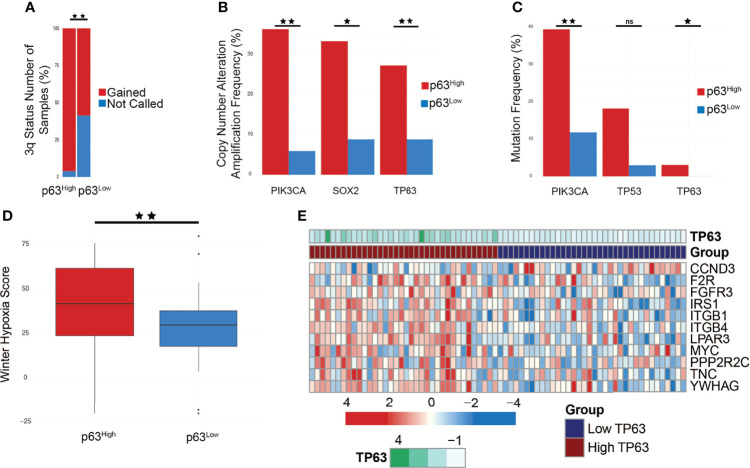
*PIK3CA* is amplified and mutated in p63^high^ tumors and affects PI3K signaling. **(A)** Bar graph comparing chromosome 3q status between p63^high^ and p63^low^ TCGA samples (*p* < 0.01). **(B)** Copy number alteration (CNA) frequencies in p63^high^ and p63^low^ TCGA samples (*PIK3CA*, *p* = 2.242e-3; *SOX2*, *p* = 0.0141; *TP63*, *p* = 0.0483). **(C)** Mutation frequencies in p63^high^ and p63^low^ TCGA samples (*PIK3CA*, *p* = 9.522e-3; *TP53*, *p* = 0.0482; *TP63*, *p* = 0.493). **(D)** Boxplot of the Winter hypoxia scores for p63^high^ and p63^low^ TCGA samples (*p* = 0.0121). **(E)** Heatmap of genes involved in PI3K signaling that were present in the combined cell line/TCGA based p63 signature. *p-value of ≤ 0.05, **p-value of ≤ 0.01, ns, non-significant.

HPV+ HNSCC subtypes also have differences in copy number alteration (CNA) patterns and mutation frequencies, specifically in *PIK3CA*. Furthermore, HPV E6 and E7 oncoproteins are implicated in regulating the PI3K/AKT/mTOR network in cancer cells under both normoxic and hypoxic conditions, specifically by regulating AKT, a main effector of both PI3K and mTORC1 signaling (71). We utilized cBioPortal to examine the genomic properties of our p63 subgroups within the TCGA HNSCC tumor dataset, and compared our findings with those by Keck et al. (14) and Zhang et al. (11). Zhang et al. (11) found that HPV-KRT tumors had more amplifications in chr3q than HPV-IMU, the subtype with a strong immune response. Notably, *TP63* is located on chr3q, providing more evidence toward p63 as a driver of this subtype. In accordance, p63^high^ samples had significantly more gains in chr3q than p63^low^ samples (**Figure 6A**). We also explored the frequency of Copy Number Alterations (CNA), specifically amplification events, of genes found within chr3q and found that *TP63*, *SOX2*, and *PIK3CA* had significantly more amplification events in the p63^high^ group (*p* = 0.0483, 0.0141, and 2.242e-3, respectively) (**Figure 6B**).

PI3K signaling plays a role in tumorigenesis, and activating mutations in *PIK3CA* have been found in various cancer types (72). Given the relationship between p63 and activated PIK3CA, we sought to explore if a p63/PI3K signaling axis is active within HPV+ HNSCC (73). First, we examined the mutational status of *PIK3CA* in the p63^high^/p63^low^ TCGA samples. Strikingly, we found that *PIK3CA* was one of the most highly mutated genes (*p* = 9.522e-3) in p63^high^ samples, with 40% of tumors harboring *PIK3CA* mutations (**Figure 6C**), similar to what has been reported for the HPV-KRT subtype. Combined with the increased copy number for *PIK3CA* in p63^high^ samples, these data strongly suggest that PI3K activity is upregulated in p63^high^ tumors. The clinical data associated with these p63^high^ and p63^low^ groups revealed a significant difference in the Winter hypoxia score, with the p63^high^ tumors having significantly higher hypoxia scores (*p* = 0.0121) (**Figure 6D**). Hypoxia stimulates AKT signaling and downregulates E6/E7 expression, inducing reversible growth arrest that is a potential pathway by which HPV+ cancers, such as HPV-KRT tumors, evade the immune response and become resistant to therapy (9, 74). The p63 signature included several PI3K pathway members that were affected by p63 knockdown in HPV+ HNSCC cell lines and were part of the p63 expression-based DEGs from the TCGA datasets (**Figure 6E**). These data point to a likely role of p63 in regulating the PI3K signaling pathway within HPV+ HNSCC.

**Figure 7:**
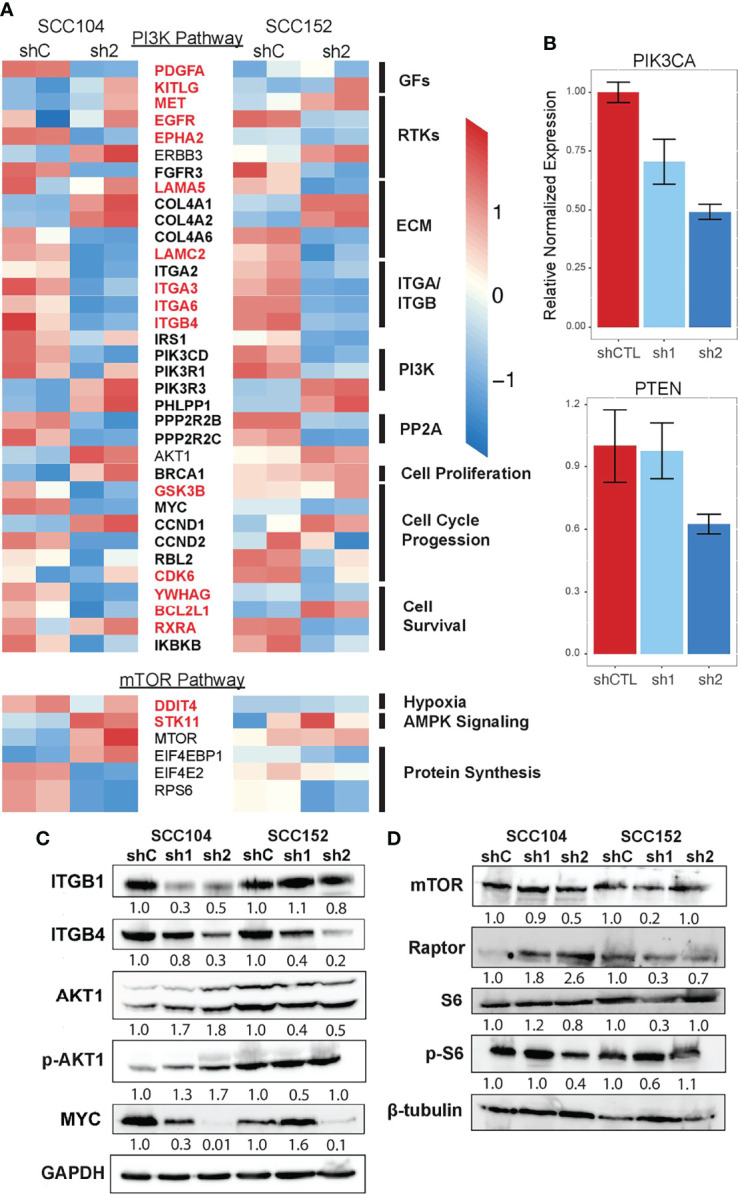
PI3K and mTOR signaling is downregulated upon loss of p63 expression. **(A)** Heatmap of select DEGs shared between SCC104 and SCC152 cells after p63 knockdown that are associated with activation and regulation of the PI3K signaling pathway. Red-labeled genes are SE marked, and bolded genes are direct p63 targets. **(B)** qRT-PCR of SCC104 shCTL and p63 shRNA cell lines. Graphs show the relative normalized expression of the catalytic subunit of PI3K as well as the PI3K phosphatase PTEN. **(C, D)** Western blot results for representative key members of the PI3K pathway **(C)** and mTOR pathway **(D)** upon p63 knockdown in SCC104 and SCC152. Densitometric analysis of protein abundance between control (shC) and knockdown (sh1 and sh2) conditions is displayed below each blot.

To systematically follow up on the p63-PI3K link, we examined the p63 knockdown datasets and found that many of the DEGs in the KEGG PI3K signaling pathway are known p63 targets, and importantly, some of these DEGs were associated with SEs (**Figure 7A**). Although the expression of many of the genes associated with PI3K signaling was decreased by p63 knockdown, the expression of *AKT1* was modestly increased. We suspect this is due to the loss of repressive effects of other p63 targets. In support of this, qRT-PCR analysis showed that expression of *PTEN*, a negative regulator of phosphorylation and activation of AKT1, is decreased upon p63 knockdown in SCC104 and SCC152 cells (**Figure 7B**). By contrast, the expression of *PIK3CA*, the gene encoding the catalytic subunit of the PI3K complex, was decreased upon knockdown of p63 (**Figure 7B**). We also examined the mTOR pathway, which is downstream of PI3K, not only because it is dysregulated in HPV+ HNSCC but also because mTOR inhibitors show promising anticancer effects in HPV+ HNSCC mouse models (75). Similar to what we found for the PI3K pathway, we saw a general loss of downstream expression of mTORC1 targets upon p63 knockdown, suggesting a downregulation of mTORC1 signaling.

Finally, we performed Western blotting for some of the proteins involved in PI3K and mTORC1 signaling to confirm the transcriptomic findings. We found that the protein levels of upstream regulators of PI3K (ITGB1 and ITGB4) were decreased in SCC104 and SCC152 cells with p63 knockdown (**Figure 7C**), in line with the RNA-seq results. A downstream target of PI3K signaling, MYC, was also downregulated by p63 knockdown, suggesting a broader dampening of PI3K signaling upon loss of p63. However, the levels of phosphorylated AKT1 (pAKT1) were elevated in SCC104 cells with p63 knockdown, suggesting the PI3K pathway is activated despite the downregulated mRNA expression of some of the key signaling components. This suggests that a complex regulatory network controls AKT activation and that other regulators (aside from p63) indirectly play a role. Western blotting also revealed mixed results for components in the mTORC1 pathway (**Figure 7D**). Whereas mTOR expression was decreased in SCC104 cells with p63 knockdown, protein levels of Raptor, a key mTOR-interacting partner, was increased (**Figure 7D**), suggesting a more complex regulatory network for mTOR activation. However, the phosphorylation of S6 ribosomal protein (pS6), a key downstream mediator of mTOR activation was reduced in SCC104, indicating that overall mTORC1 signaling was dampened by the loss of p63 (**Figure 7D**). Taken together, these results point to a complex signaling network by which p63 regulates the PI3K and mTORC1 signaling pathway in HPV+ HNSCC, which may have implications for potential therapeutics.

## Discussion

HNSCC associated with high-risk human HPV infection is a growing problem that is clinically and biologically distinct from HPV- HNSCC. Molecular studies of HPV+ HNSCC in the past focused primarily on tumor suppressor pathways that are targeted by viral oncoproteins such as E6, which inactivates the p53 tumor suppressor protein by instigating its degradation (9). However, the operation of oncogenic drivers in the underlying complex genomic and epigenomic milieu of HPV+ HNSCC remains unclear. Studies of p63, especially oncogenic ΔNp63, have established its role in directing broad transcriptional programs in SCCs in various anatomical sites, including the oral cavity (21, 49, 76). However, the specific role of p63 in modulating the transcriptomic landscape of HPV+ HNSCC has received less attention. To address this shortcoming, we leveraged genomic, transcriptomic, and epigenomic data from HPV+ HNSCC tumors and preclinical cell line models and identified p63 as a critical regulator that affects multiple facets of HPV+ HNSCC biology, including pathways essential in HPV-mediated carcinogenesis, and HNSCC subtype-specific gene expression.

One notable finding is the wide range of p63 expression levels across the HPV+ tumors, which is clearly evident in several independent datasets. This reinforces results from previously defined subtypes of HPV+ HNSCC based on hierarchical clustering of gene expression (11, 13, 14, 51). We provide evidence that the molecular and phenotypic attributes of the more aggressive p63^high^ HPV-KRT subtype, such as keratinization and cell adhesion, are likely controlled by a p63-driven direct transcriptional program. Indeed, this is well supported by our integrated analysis of several complementary models that include HPV+ patient tumors and by an epigenetically refined ΔNp63 cistrome assembled from RNA-seq after p63 knockdown and ChIP-seq of p63 and histone marks in SCC104 and SCC152 cell lines. We suspect that the heterogeneity of HPV+ tumors is shaped not only by the expression and the transcriptional output of p63 in the tumor epithelium but also by the enhanced influence of p63 in the tumor microenvironment, as evidenced in triple-negative breast cancer (77). This notion is supported by our identification of enriched immune pathways and immune-related genes as p63 targets, some of which were associated with SEs in SCC104 and SCC152 cells.

The tumor microenvironment of HPV+ HNSCC is distinct from that of its HPV- counterpart, with greater immune infiltration, T-cell activation, and immunoregulation (51). Furthermore, two recent landmark single-cell-based studies indicated that HPV-specific infiltrating lymphocytes may mount an immune-based response to HPV+ HNSCC (78, 79). In these studies, HPV oncoprotein E2 was a target of particular interest, as E2 expression is often maintained in HPV+ HNSCC unlike in HPV+ cervical cancer. Interestingly, E2 expression is lower in the p63^high^ HPV-KRT subtype, most likely because of higher levels of HPV genomic integration, which disrupts HPV early gene expression (11, 51). We suspect that the relatively lower immune cell infiltration and activation in HPV-KRT tumors may be attributable to their p63^high^ and E2^low^ state. Taken together, these studies suggest that more attention should be paid to HPV+ subtypes, because their inherent differences, especially in their tumor microenvironments, likely affect their clinical outcomes.

Our integrated analysis revealed many interesting insights into p63 and its link to specific aspects of HPV biology. One novel potential regulatory mechanism of p63 involves E2F family members, notably E2F1 and E2F7, which regulate cell cycle gene expression in HPV infection and HPV-associated cancers (10, 80). Indeed, E2F7 was consistently enriched throughout our analyses as a direct target of p63 and as a TF whose motif was enriched around many p63-bound genomic sites and in SEs. E2F7 is an atypical E2F factor and a transcriptional repressor of genes involved in genomic stability, cell proliferation, and migration (80, 81). Knockdown of p63 in HPV+ HNSCC cell lines upregulated E2F7 expression, leading us to speculate that the increased p63 expression in HPV+ HNSCC negates the repressive function of E2F7. It is possible that p63 and E2F7 physically interact and coregulate genes that have joint p63/E2F motifs in their regulatory elements, an interesting notion that could be experimentally tested pending the availability of ChIP-grade E2F7 antibodies. Further investigation into the p63-E2F network is needed given the p63-dependent enrichment of pathways related to pRB, the cell cycle, and cell cycle checkpoints that are relevant for HPV+ HNSCC. Along the same line, it is likely that p63-p53 interactions are a key component in the cell cycle circuitry given p63’s ability to regulate some p53 targets in the presence or absence of p53 (15, 82, 83).

The results from our studies reemphasize the need to further characterize the drivers and molecular attributes of HPV+ HNSCC subtypes given the potential differences in overall patient survival between the subtypes. This is particularly important in light of recent efforts to identify treatment de-escalation strategies for HPV+ HNSCC to reduce adverse events while maintaining better oncologic outcomes. Unfortunately, two large phase III clinical trials have shown inferior overall survival and progression-free survival as well as increased rates of locoregional failure (6, 84, 85), prompting reevaluation of ongoing deintensification trials for HNSCC. Although current HNSCC treatment options take HPV status into account, we posit that a personalized genomics approach that considers HPV+ subtypes would better inform treatment options and prevent failure of treatment de-escalation. The activated PI3K signaling that we uncovered in the p63^high^ HPV-KRT subtype also suggests a potential avenue for therapeutic intervention. Compensatory activation of downstream signaling pathways, including PI3K, has been suggested as one of the major mechanisms of resistance to EGFR inhibitors, including cetuximab (86). The addition of a mTOR/PI3K inhibitor effectively controls cell growth in EGFR inhibitor-resistant HNSCC, suggesting that combination therapy may increase treatment efficacy (87, 88). In addition, mTOR inhibitors show promising anticancer effects in HPV+ HNSCC xenograft mouse models (75). We suspect that patients with the p63^high^ tumor subtype would benefit from a combination of EGFR and PI3K inhibitor treatment and radiotherapy.

Altogether, the results from our studies suggest p63 and its key downstream effectors can be used as stratification markers for HPV+ HNSCC patients. However, this requires validation of our preclinical genomic and epigenomic data in tumors from HPV+ HNSCC patients.

## Data Availability Statement

The datasets presented in this study can be found in online repositories. The names of the repository/repositories and accession number(s) can be found below: https://www.ncbi.nlm.nih.gov/geo/query/acc.cgi?acc=GSE182133.

## Author Contributions

AG designed and performed experiments, acquired and analyzed data, prepared the figures, and wrote the manuscript. AO and CG performed experiments and acquired data. JB analyzed data. SS supervised the project and experimental design and wrote the manuscript. All authors contributed to the article and approved the submitted version.

## Funding

AG was supported partly by NYSTEM contract no. C30290GG.

## Conflict of Interest

The authors declare that the research was conducted in the absence of any commercial or financial relationships that could be construed as a potential conflict of interest.

## Publisher’s Note

All claims expressed in this article are solely those of the authors and do not necessarily represent those of their affiliated organizations, or those of the publisher, the editors and the reviewers. Any product that may be evaluated in this article, or claim that may be made by its manufacturer, is not guaranteed or endorsed by the publisher.

## References

[1] Ferlay J Shin HR Bray F Forman D Mathers C Parkin DM. Estimates of Worldwide Burden of Cancer in 2008: GLOBOCAN 2008. Int J Cancer (2010) 127(12):2893–917. doi: 10.1002/ijc.25516 21351269

[2] Marur S D’Souza G Westra WH Forastiere AA. HPV-Associated Head and Neck Cancer: A Virus-Related Cancer Epidemic – A Review of Epidemiology, Biology, Virus Detection and Issues in Management. Lancet Oncol (2020) 11(3):781–9. doi: 10.1016/S1470-2045(10)70017-6 PMC5242182 20451455

[3] Seiwert TY Zuo Z Keck MK Khattri A Pedamallu CS Stricker T. Integrative and Comparative Genomic Analysis of HPV-Positive and HPV-Negative Head and Neck Squamous Cell Carcinomas. Clin Cancer Res (2015) 21(3):632–41. doi: 10.1158/1078-0432.CCR-13-3310 PMC4305034 25056374

[4] Leemans CR Snijders PJF Brakenhoff RH. The Molecular Landscape of Head and Neck Cancer. Nat Rev Cancer (2018) 18(5):269–82. doi: 10.1038/nrc.2018.11 29497144

[5] Dok R Nuyts S. HPV Positive Head and Neck Cancers: Molecular Pathogenesis and Evolving Treatment Strategies. Cancers (Basel) (2016) 8(41):1–16. doi: 10.3390/cancers8040041 PMC4846850 27043631

[6] Ventz S Trippa L Schoenfeld JD. Lessons Learned From Deescalation Trials in Favorable Risk HPV-Associated Squamous Cell Head and Neck Cancer–A Perspective on Future Trial Designs. Clin Cancer Res (2019) 25(24):7281–6. doi: 10.1158/1078-0432.CCR-19-0945 31527164

[7] Groves IJ Coleman N. Human Papillomavirus Genome Integration in Squamous Carcinogenesis: What Have Next-Generation Sequencing Studies Taught Us? J Pathol (2018) 245(1):9–18. doi: 10.1002/path.5058 29443391

[8] Viarisio D Gissmann L Tommasino M. Human Papillomaviruses and Carcinogenesis: Well-Established and Novel Models. Curr Opin Virol (2017) 26:56–62. doi: 10.1016/j.coviro.2017.07.014 28778034

[9] Hoppe-Seyler K Bossler F Braun JA Herrmann AL Hoppe-Seyler F. The HPV E6/E7 Oncogenes: Key Factors for Viral Carcinogenesis and Therapeutic Targets. Trends Microbiol (2018) 26(2):158–68. doi: 10.1016/j.tim.2017.07.007 28823569

[10] Faraji F Zaidi M Fakhry C Gaykalova DA. Molecular Mechanisms of Human Papillomavirus-Related Carcinogenesis in Head and Neck Cancer. Microbes Infect (2017) 19(9–10):464–75. doi: 10.1016/j.micinf.2017.06.001 PMC5603399 28619685

[11] Zhang Y Koneva LA Virani S Arthur AE Virani A Hall PB. Subtypes of HPV-Positive Head and Neck Cancers Are Associated With HPV Characteristics, Copy Number Alterations, PIK3CA Mutation, and Pathway Signatures. Clin Cancer Res (2016) 22(18):4735–45. doi: 10.1158/1078-0432.CCR-16-0323 PMC5026546 27091409

[12] Walline HM Goudsmit CM McHugh JB Tang AL Owen JH Teh BT. Integration of High-Risk Human Papillomavirus Into Cellular Cancer-Related Genes in Head and Neck Cancer Cell Lines. Head Neck (2017) 39(5):840–52. doi: 10.1002/hed.24729 PMC5392184 28236344

[13] Walter V Yin X Wilkerson MD Cabanski CR Zhao N Du Y. Molecular Subtypes in Head and Neck Cancer Exhibit Distinct Patterns of Chromosomal Gain and Loss of Canonical Cancer Genes. PloS One (2013) 8(2):e56823. doi: 10.1371/journal.pone.0056823 23451093 PMC3579892

[14] Keck MK Zuo Z Khattri A Stricker TP Brown CD Imanguli M. Integrative Analysis of Head and Neck Cancer Identifies Two Biologically Distinct HPV and Three Non-HPV Subtypes. Clin Cancer Res (2015) 21(4):870–81. doi: 10.1158/1078-0432.CCR-14-2481 25492084

[15] Botchkarev V Flores E. P53/P63/P73 in the Epidermis in Health and Disease. Cold Spring Harb Perspect Med (2014) 4(8):1260–6. doi: 10.1101/cshperspect.a015248 PMC4109579 25085956

[16] Romano R Smalley K Magraw C Serna VA Kurita T Raghavan S. Np63 Knockout Mice Reveal Its Indispensable Role as a Master Regulator of Epithelial Development and Differentiation. Development (2012) 139(4):772–82. doi: 10.1242/dev.071191 PMC3265062 22274697

[17] Romano R Ortt K Birkaya B Smalley K Sinha S. An Active Role of the D N Isoform of P63 in Regulating Basal Keratin Genes K5 and K14 and Directing Epidermal Cell Fate. PloS One (2009) 4(5):e5623. doi: 10.1371/journal.pone.0005623 19461998 PMC2680039

[18] Soares E Zhou H. Master Regulatory Role of P63 in Epidermal Development and Disease. Cell Mol Life Sci (2018) 75(7):1179–90. doi: 10.1007/s00018-017-2701-z PMC5843667 29103147

[19] Sethi I Romano RA Gluck C Smalley K Vojtesek B Buck MJ. A Global Analysis of the Complex Landscape of Isoforms and Regulatory Networks of P63 in Human Cells and Tissues. BMC Genomics (2015) 16(1):1–15. doi: 10.1186/s12864-015-1793-9 26251276 PMC4528692

[20] Abbas HA Bui NHB Rajapakshe K Wong J Gunaratne P Tsai KY. Distinct TP63 Isoform-Driven Transcriptional Signatures Predict Tumor Progression and Clinical Outcomes. Cancer Res (2018) 78(2):451–62. doi: 10.1158/0008-5472.CAN-17-1803 PMC5771893 29180475

[21] Moses MA George AL Sakakibara N Mahmood K Ponnamperuma RM King KE. Molecular Mechanisms of P63-Mediated Squamous Cancer Pathogenesis. Int J Mol Sci (2019) 20:1–21. doi: 10.3390/ijms20143590 PMC6678256 31340447

[22] Rocco JW Leong CO Kuperwasser N DeYoung MP Ellisen LW. P63 Mediates Survival in Squamous Cell Carcinoma By Suppression of P73-Dependent Apoptosis. Cancer Cell (2006) 9(1):45–56. doi: 10.1016/j.ccr.2005.12.013 16413471

[23] Citro S Bellini A Medda A Sabatini ME Tagliabue M Chu F. Human Papilloma Virus Increases Δnp63α Expression in Head and Neck Squamous Cell Carcinoma. Front Cell Infect Microbiol (2020) 10(April):1–6. doi: 10.3389/fcimb.2020.00143 32322564 PMC7156594

[24] Tang AL Hauff SJ Owen JH Graham MP Czerwinkski MJ Park JJ. UM-SCC-104: A New Human Papillomavirus-16–Positive Cancer Stem Cell–Containing Head and Neck Squamous Cell Carcinoma Cell Line. Head Neck (2011) 34(10):1480–91. doi: 10.1002/hed.21962 PMC3369005 22162267

[25] Greaney-Davies FST Risk JM Robinson M Liloglou T Shaw RJ Schache AG. Essential Characterisation of Human Papillomavirus Positive Head and Neck Cancer Cell Lines. Oral Oncol (2020) 103:104613. doi: 10.1016/j.oraloncology.2020.104613 32120342

[26] Love MI Huber W Anders S. Moderated Estimation of Fold Change and Dispersion for RNA-Seq Data With Deseq2. Genome Biol (2014) 15(12):1–21. doi: 10.1186/s13059-014-0550-8 PMC4302049 25516281

[27] Gluck C Glathar A Tsompana M Nowak N Garrett-Sinha LA Buck MJ. Molecular Dissection of the Oncogenic Role of ETS1 in the Mesenchymal Subtypes of Head and Neck Squamous Cell Carcinoma. PloS Genet (2019) 15(7):1–31. doi: 10.1371/journal.pgen.1008250 PMC6657958 31306413

[28] Nekulova M Holcakova J Nenutil R Stratmann R Bouchalova P Müller P. Characterization of Specific P63 and P63-N-Terminal Isoform Antibodies and Their Application for Immunohistochemistry. Virchows Arch (2013) 463(3):415–25. doi: 10.1007/s00428-013-1459-4 23887585

[29] Langmead B Trapnell C Pop M Salzberg SL. Ultrafast and Memory-Efficient Alignment of Short DNA Sequences to the Human Genome. Genome Biol (2009) 10(3):1–10. doi: 10.1186/gb-2009-10-3-r25 PMC2690996 19261174

[30] Zhang Y Liu T Meyer CA Eeckhoute J Johnson DS Bernstein BE. Model-Based Analysis of ChIP-Seq (MACS). Genome Biol (2008) 9(9):1–9. doi: 10.1186/gb-2008-9-9-r137 PMC2592715 18798982

[31] Mclean CY Bristor D Hiller M Clarke SL Schaar BT Wenger AM. GREAT Improves Functional Interpretation of Cis-Regulatory Regions. Nat Biotechnol (2010) 28(5):495–501. doi: 10.1038/nbt.1630 20436461 PMC4840234

[32] Ramírez F Ryan DP Grüning B Bhardwaj V Kilpert F Richter AS. Deeptools2: A Next Generation Web Server for Deep-Sequencing Data Analysis. Nucleic Acids Res (2016) 44(W1):W160–5. doi: 10.1093/nar/gkw257 PMC4987876 27079975

[33] Heinz S Benner C Spann N Bertolino E Lin YC Laslo P. Simple Combinations of Lineage-Determining Transcription Factors Prime Cis-Regulatory Elements Required for Macrophage and B Cell Identities. Mol Cell (2010) 38(4):576–89. doi: 10.1016/j.molcel.2010.05.004 PMC2898526 20513432

[34] Kim D Paggi JM Park C Bennett C Salzberg SL. Graph-Based Genome Alignment and Genotyping With HISAT2 and HISAT-Genotype. Nat Biotechnol (2019) 37(8):907–15. doi: 10.1038/s41587-019-0201-4 PMC7605509 31375807

[35] Wagner GP Kin K Lynch VJ. Measurement of mRNA Abundance Using RNA-Seq Data: RPKM Measure Is Inconsistent Among Samples. Theory Biosci (2012) 131(4):281–5. doi: 10.1007/s12064-012-0162-3 22872506

[36] Ando M Saito Y Xu G Bui NQ Medetgul-Ernar K Pu M. Chromatin Dysregulation and DNA Methylation at Transcription Start Sites Associated With Transcriptional Repression in Cancers. Nat Commun (2019) 10(1):1–15. doi: 10.1038/s41467-019-09937-w 31097695 PMC6522544

[37] Wood O Woo J Seumois G Savelyeva N McCann KJ Singh D. Gene Expression Analysis of TIL Rich HPV-Driven Head and Neck Tumors Reveals a Distinct B-Cell Signature When Compared to HPV Independent Tumors. Oncotarget (2016) 7(35):56781–97. doi: 10.18632/oncotarget.10788 PMC5302866 27462861

[38] Gleber-netto FO Skinner HD Curtis R Gleber-netto FO Rao X Guo T. Variations in HPV Function are Associated With Survival in Squamous Cell Carcinoma. JCI Insight (2019) 4(1):e124762. doi: 10.1172/jci.insight.124762 PMC6485353 30626753

[39] Cerami E Gao J Dogrusoz U Gross BE Sumer SO Aksoy BA. The CBio Cancer Genomics. Cancer Discovery (2017) 32(7):736–40. doi: 10.1158/2159-8290.CD-12-0095 PMC3956037 22588877

[40] Gao J Aksoy BA Dogrusoz U Dresdner G Gross B Sumer SO. Integrative Analysis of Complex Cancer Genomics and Clinical Profiles Using the Cbioportal Complementary Data Sources and Analysis Options. Sci Signal (2014) 6(269):1–20. doi: 10.1126/scisignal.2004088 PMC4160307 23550210

[41] Whyte WA Orlando DA Hnisz D Abraham BJ Lin CY Kagey MH. Master Transcription Factors and Mediator Establish Super- Enhancers at Key Cell Identity Genes. Cell (2013) 153(2):307–19. doi: 10.1016/j.cell.2013.03.035 PMC3653129 23582322

[42] Loven J Hoke HA Lin CY Lau A Orlando DA Vakoc CR. Selective Inhibition of Tumor Oncogenes by Disruption of Super-Enhancers. Cell (2013) 153:320–34. doi: 10.1016/j.cell.2013.03.036 PMC3760967 23582323

[43] Georgiou G van Heeringen SJ. Fluff: Exploratory Analysis and Visualization of High-Throughput Sequencing Data. PeerJ (2016) 2016(7):1–10. doi: 10.1101/045526 PMC4957989 27547532

[44] Shin H Liu T Manrai AK Liu SX. CEAS: Cis-Regulatory Element Annotation System. Bioinformatics (2009) 25(19):2605–6. doi: 10.1093/bioinformatics/btp479 19689956

[45] Kanehisa M Furumichi M Sato Y Ishiguro-Watanabe M Tanabe M. KEGG: Integrating Viruses and Cellular Organisms. Nucleic Acids Res (2021) 49(D1):D545–51. doi: 10.1093/nar/gkaa970 PMC7779016 33125081

[46] Huang DW Sherman BT Lempicki RA. Systematic and Integrative Analysis of Large Gene Lists Using DAVID Bioinformatics Resources. Nat Protoc (2009) 4(1):44–57. doi: 10.1038/nprot.2008.211 19131956

[47] Huang DW Sherman BT Lempicki RA. Bioinformatics Enrichment Tools: Paths Toward the Comprehensive Functional Analysis of Large Gene Lists. Nucleic Acids Res (2009) 37(1):1–13. doi: 10.1093/nar/gkn923 19033363 PMC2615629

[48] Subramanian A Tamayo P Mootha VK Mukherjee S Ebert BL Gillette MA. Gene Set Enrichment Analysis: A Knowledge-Based Approach for Interpreting Genome-Wide Expression Profiles. Proc Natl Acad Sci USA (2005) 102(43):15545–50. doi: 10.1073/pnas.0506580102 PMC1239896 16199517

[49] Gatti V Fierro C Annicchiarico-Petruzzelli M Melino G Peschiaroli A. Δnp63 in Squamous Cell Carcinoma: Defining the Oncogenic Routes Affecting Epigenetic Landscape and Tumour Microenvironment. Mol Oncol (2019) 13(5):981–1001. doi: 10.1002/1878-0261.12473 30845357 PMC6487733

[50] Lo Muzio L Santarelli A Caltabiano R Rubini C Pieramici T Trevisiol L. P63 Overexpression Associates With Poor Prognosis in Head and Neck Squamous Cell Carcinoma. Hum Pathol (2005) 36(2):187–94. doi: 10.1016/j.humpath.2004.12.003 15754296

[51] Qin T Li S Henry LE Liu S Sartor MA. Molecular Tumor Subtypes of Hpv-Positive Head and Neck Cancers: Biological Characteristics and Implications for Clinical Outcomes. Cancers (Basel) (2021) 13(11):1–21. doi: 10.3390/cancers13112721 PMC8198180 34072836

[52] Si H Lu H Yang X Mattox A Jang M Bian Y. TNF-α Modulates Genome-Wide Redistribution of Np63α/TAp73 and NF-κb C-REL Interactive Binding on TP53 and AP-1 Motifs to Promote an Oncogenic Gene Program in Squamous Cancer. Oncogene (2016) 35(44):5781–94. doi: 10.1038/onc.2016.112 PMC5093089 27132513

[53] Budhwani M Lukowski SW Porceddu SV Frazer IH Chandra J. Dysregulation of Stemness Pathways in HPV Mediated Cervical Malignant Transformation Identifies Potential Oncotherapy Targets. Front Cell Infect Microbiol (2020) 10(June):1–9. doi: 10.3389/fcimb.2020.00307 32670895 PMC7330094

[54] Kivi N Greco D Auvinen P Auvinen E. Genes Involved in Cell Adhesion, Cell Motility and Mitogenic Signaling Are Altered Due to HPV 16 E5 Protein Expression. Oncogene (2008) 27(18):2532–41. doi: 10.1038/sj.onc.1210916 17982485

[55] Poreba E Broniarczyk JK Gozdzicka-Jozefiak A. Epigenetic Mechanisms in Virus-Induced Tumorigenesis. Clin Epigenet (2011) 2(2):233–47. doi: 10.1007/s13148-011-0026-6 PMC3365383 22704339

[56] Heinz S Romanoski CE Benner C Glass CK. The Selection and Function of Cell Type-Specific Enhancers. Nat Rev Mol Cell Biol (2015) 16(3):144–54. doi: 10.1038/nrm3949 PMC4517609 25650801

[57] Sigova AA Hnisz D Abraham BJ Lee TI Lau A Saint-andre V. Super-Enhancers in the Control of Cell Identity and Disease. Cell (2013) 155:934–47. doi: 10.1016/j.cell.2013.09.053 PMC3841062 24119843

[58] Patel D Huang SM Baglia LA McCance DJ. The E6 Protein of Human Papillomavirus Type 16 Binds to and Inhibits Co-Activation by CBP and P300. EMBO J (1999) 18(18):5061–72. doi: 10.1093/emboj/18.18.5061 PMC1171577 10487758

[59] Veldman T Liu X Yuan H Schlegel R. Human Papillomavirus E6 and Myc Proteins Associate *In Vivo* and Bind to and Cooperatively Activate the Telomerase Reverse Transcriptase Promoter. Proc Natl Acad Sci USA (2003) 100(14):8211–6. doi: 10.1073/pnas.1435900100 PMC166208 12821782

[60] Dooley KE Warburton A McBride AA. Tandemly Integrated HPV16 can Form a Brd4-Dependent Super-Enhancer-Like Element That Drives Transcription of Viral Oncogenes. MBio (2016) 7(5):1–10. doi: 10.1128/mBio.01446-16 PMC5021809 27624132

[61] Whyte WA Orlando DA Hnisz D Abraham BJ Lin CY Kagey MH. Master Transcription Factors and Mediator Establish Super-Enhancers at Key Cell Identity Genes. Cell (2013) 153(2):307–19. doi: 10.1016/j.cell.2013.03.035 PMC3653129 23582322

[62] Bello JOM Nieva LO Paredes AC Gonzalez AMF Zavaleta LR Lizano M. Regulation of the Wntβ-Catenin Signaling Pathway by Human Papillomavirus E6 and E7 Oncoproteins. Viruses (2015) 7(8):4734–55. doi: 10.3390/v7082842 PMC4576203 26295406

[63] Khelil M Griffin H Bleeker MCG Steenbergen RDM Zheng K Saunders-Wood T. Delta-Like Ligand-Notch1 Signalling Is Selectively Modulated by HPV16 E6 to Promote Squamous Cell Proliferation and Correlates With Cervical Cancer Prognosis. Cancer Res (2021) 81(7):1909–21. doi: 10.1158/0008-5472.CAN-20-1996 33500246

[64] Zhang X Wu J Luo S Lechler T Zhang JY. FRA1 Promotes Squamous Cell Carcinoma Growth and Metastasis Through Distinct AKT and C-Jun Dependent Mechanisms. Oncotarget (2016) 7(23):34371–83. doi: 10.18632/oncotarget.9110 PMC5085162 27144339

[65] Surviladze Z Sterkand RT Ozbun MA. Interaction of Human Papillomavirus Type 16 Particles With Heparan Sulfate and Syndecan-1 Molecules in the Keratinocyte Extracellular Matrix Plays an Active Role in Infection. J Gen Virol (2015) 96(8):2232–41. doi: 10.1099/vir.0.000147 PMC4681067 26289843

[66] D’Costa ZJ Jolly C Androphy EJ Mercer A Matthews CM Hibma MH. Transcriptional Repression of E-Cadherin by Human Papillomavirus Type 16 E6. PloS One (2012) 7(11):e48954. doi: 10.1371/journal.pone.0048954 23189137 PMC3506579

[67] Alzahrani F Clattenburg L Muruganandan S Bullock M MacIsaac K Wigerius M. The Hippo Component YAP Localizes in the Nucleus of Human Papilloma Virus Positive Oropharyngeal Squamous Cell Carcinoma. J Otolaryngol Head Neck Surg (2017) 46(1):1–7. doi: 10.1186/s40463-017-0187-1 28222762 PMC5320711

[68] Sundqvist A Vasilaki E Voytyuk O Bai Y Morikawa M Moustakas A. Tgfβ and EGF Signaling Orchestrates the AP-1- and P63 Transcriptional Regulation of Breast Cancer Invasiveness. Oncogene (2020) 39(22):4436–49. doi: 10.1038/s41388-020-1299-z PMC7253358 32350443

[69] Lakshmanachetty S Balaiya V High WA Koster MI. Loss of TP63 Promotes the Metastasis of Head and Neck Squamous Cell Carcinoma by Activating MAPK and STAT3 Signaling. Mol Cancer Res (2019) 17(6):1–16. doi: 10.1158/1541-7786.MCR-18-1355 PMC6548645 30910837

[70] Chakrabarti R Romano R-A Kannan N Amadori D Choudhury A Hang X. Δnp63 Promotes Stem Cell Activity in Mammary Gland Development and Basal-Like Breast Cancer by Enhancing Fzd7 Expression and Wnt Signaling. Nat Cell Biol (2014) 16(10):1004–15. doi: 10.1038/ncb3040 PMC4183725 25241036

[71] Bossler F Hoppe-Seyler K Hoppe-Seyler F. PI3K/AKT/mTOR Signaling Regulates the Virus/Host Cell Crosstalk in HPV-Positive Cervical Cancer Cells. Int J Mol Sci (2019) 20(9):1–13. doi: 10.3390/ijms20092188 PMC6539191 31058807

[72] Yuan TL Cantley LC. PI3K Pathway Alterations in Cancer: Variations on a Theme. Oncogene (2008) 27(41):5497–510. doi: 10.1038/onc.2008.245 PMC3398461 18794884

[73] Hu L Liang S Chen H Lv T Wu J Chen D. δnp63α is a Common Inhibitory Target in Oncogenic PI3K/Ras/Her2-Induced Cell Motility and Tumor Metastasis. Proc Natl Acad Sci (2017) 114(20):E3964–73. doi: 10.1073/pnas.1617816114 28468801 PMC5441775

[74] Bossler F Kuhn BJ Gunther T Kraemer SJ Khalkar P Adrian S. Repression of Human Papillomavirus Oncogene Expression Under Hypoxia Is Mediated by PI3K/mTORC2/AKT Signaling. MBio (2019) 10(1):1–16. doi: 10.1128/mBio.02323-18 PMC6372795 30755508

[75] Molinolo AA Marsh C El-Dinali M Gangane N Jennison K Hewitt S. mTOR as a Molecular Target in HPV-Associated Oral and Cervical Squamous Carcinomas. Clin Cancer Res (2012) 18(9):2558–68. doi: 10.1158/1078-0432.CCR-11-2824 PMC3443560 22409888

[76] Ramsey MR Wilson C Ory B Rothenberg SM Faquin W Mills AA. FGFR2 Signaling Underlies P63 Oncogenic Function in Squamous Cell Carcinoma. J Clin Invest (2013) 123(8):3525–38. doi: 10.1172/JCI68899 PMC3726171 23867503

[77] Kumar S Gabrilovich D Chakrabarti R Kumar S Wilkes DW Samuel N. Δnp63-Driven Recruitment of Myeloid-Derived Suppressor Cells Promotes Metastasis in Triple- Negative Breast Cancer. J Clin Invest (2018) 128(11):5095–109. doi: 10.1172/JCI99673 PMC6205409 30295647

[78] Wieland A Patel MR Cardenas MA Eberhardt CS Hudson WH Obeng RC. Defining HPV-Specific B Cell Responses in Patients With Head and Neck Cancer. Nature (2021) 597:274–8. doi: 10.1038/s41586-020-2931-3 PMC9462833 33208941

[79] Eberhardt CS Kissick HT Patel MR Cardenas MA Prokhnevska N Obeng RC. Functional HPV-Specific PD-1+ Stem-Like CD8 T Cells in Head and Neck Cancer. Nature (2021) 597(7875):279–84. doi: 10.1038/s41586-021-03862-z PMC10201342 34471285

[80] Mitxelena J Apraiz A Vallejo-Rodríguez J García-Santisteban I Fullaondo A Alvarez-Fernández M. An E2F7-Dependent Transcriptional Program Modulates DNA Damage Repair and Genomic Stability. Nucleic Acids Res (2018) 46(9):4546–59. doi: 10.1093/nar/gky218 PMC5961008 29590434

[81] Yang C Zhang ZC Liu TB Xu Y Xia BR Lou G. E2F1/2/7/8 as Independent Indicators of Survival in Patients With Cervical Squamous Cell Carcinoma. Cancer Cell Int (2020) 20(1):1–17. doi: 10.1186/s12935-020-01594-0 33061852 PMC7552358

[82] Mighty KK Laimins LA. P63 Is Necessary for the Activation of Human Papillomavirus Late Viral Functions Upon Epithelial Differentiation. J Virol (2011) 85(17):8863–9. doi: 10.1128/JVI.00750-11 PMC3165790 21715473

[83] Dohn M Zhang S Chen X. P63α and Δnp63α can Induce Cell Cycle Arrest and Apoptosis and Differentially Regulate P53 Target Genes. Oncogene (2001) 20(25):3193–205. doi: 10.1038/sj.onc.1204427 11423969

[84] Gillison ML Trotti AM Harris J Eisbruch A Harari PM Adelstein DJ. Radiotherapy Plus Cetuximab or Cisplatin for Human Papillomavirus (HPV)-Positive Oropharyngeal Cancer: A Randomized, Multicenter, non-Inferiority Clinical Trial. Lancet (2019) 393(10166):40–50. doi: 10.1016/S0140-6736(18)32779-X 30449625 PMC6541928

[85] Mehanna H Robinson M Hartley A Kong A Foran B Fulton-Lieuw T. Radiotherapy Plus Cisplatin or Cetuximab in Low-Risk Human Papillomavirus-Positive Oropharyngeal Cancer (De-ESCALaTE HPV): An Open-Label Randomised Controlled Phase 3 Trial. Lancet (2019) 393(10166):51–60. doi: https://doi.org/10.1016/S0140-6736(18)32752-1 30449623 10.1016/S0140-6736(18)32752-1 PMC6319250

[86] Keysar SB Astling DP Anderson RT Vogler BW Bowles DW Morton JJ. A Patient Tumor Transplant Model of Squamous Cell Cancer Identifies PI3K Inhibitors as Candidate Therapeutics in Defined Molecular Bins. Mol Oncol (2013) 7(4):776–90. doi: 10.1016/j.molonc.2013.03.004 PMC3760013 23607916

[87] Wang Z Martin D Molinolo AA Patel V Iglesias-Bartolome R Degese MS. MTOR Co-Targeting in Cetuximab Resistance in Head and Neck Cancers Harboring PIK3CA and RAS Mutations. J Natl Cancer Inst (2014) 106(9):1–11. doi: 10.1093/jnci/dju215 PMC4133928 25099740

[88] D’Amato V Rosa R D’Amato C Formisano L Marciano R Nappi L. The Dual PI3K/mTOR Inhibitor PKI-587 Enhances Sensitivity to Cetuximab in EGFR-Resistant Human Head and Neck Cancer Models. Br J Cancer (2014) 110(12):2887–95. doi: 10.1038/bjc.2014.241 PMC4056056 24823695

