# Supplementary material for: p63 Directs Subtype-Specific Gene Expression in HPV+ Head and Neck Squamous Cell Carcinoma
Source: Front Oncol. 2022 May 31;12:879054. doi: 10.3389/fonc.2022.879054 (PMC9192977; doi:10.3389/fonc.2022.879054)
Supplement: Supplementary file 1 [file DataSheet_1.pdf]

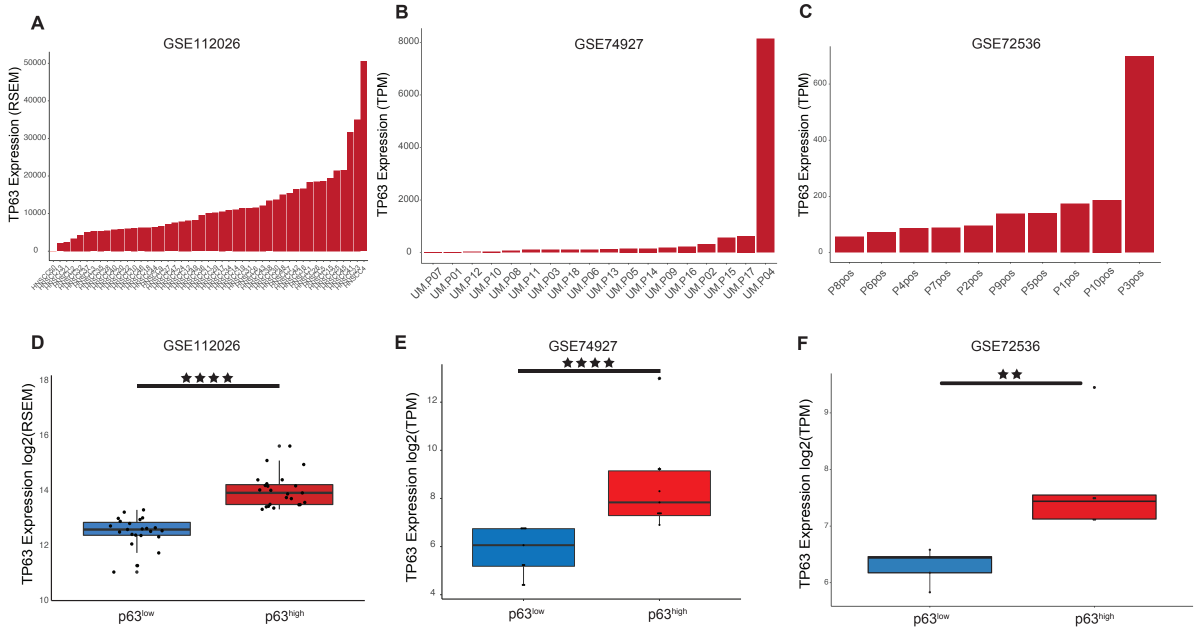

Supplementary Figure 1: There is a gradient of p63 expression across HPV+ HNSCC tumors. (A) Bar chart of p63 expression across tumors in the GSE112026 dataset. Underneath, tumors were segregated into $p63^{low}$ and $p63^{high}$ groups based on median p63 expression. Boxplots display the significant difference in expression of *TP63* across the groups (p-value - 1.24e-13). **(B)** Bar chart of p63 expression across tumors in the GSE74927 dataset. Underneath, tumors were segregated into $p63^{low}$ and $p63^{high}$ groups based on median p63 expression. Boxplots display the significant difference in expression of *TP63* across the groups (p-value - 4.048e-5). **(C)** Bar chart of p63 expression across tumors in the GSE72536 dataset. Underneath, tumors were segregated into $p63^{low}$ and $p63^{high}$ groups based on median p63 expression. Boxplots display the significant difference in expression of *TP63* across the groups (p-value - 0.0079).

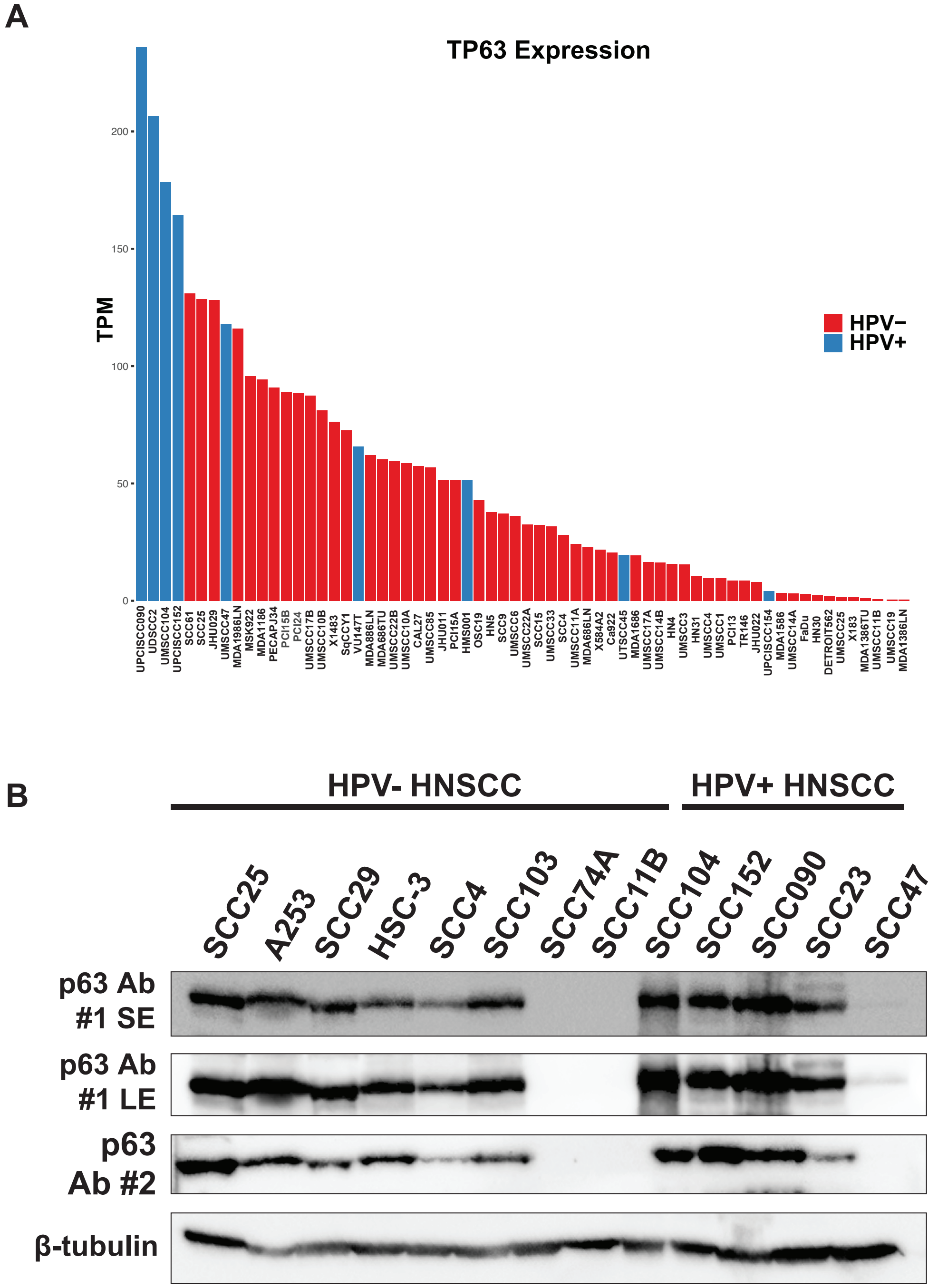

Supplementary Figure 2: Expression of p63 across HNSCC HPV+ and HPV- cell lines. **(A)** Expression of p63 across HPV+ and HPV- cell lines from the Gleber-Netto dataset (GSE122512) (27). **(B)** Western blot analysis of p63 expression across HNSCC cell lines (8 HPV- and 5 HPV+).

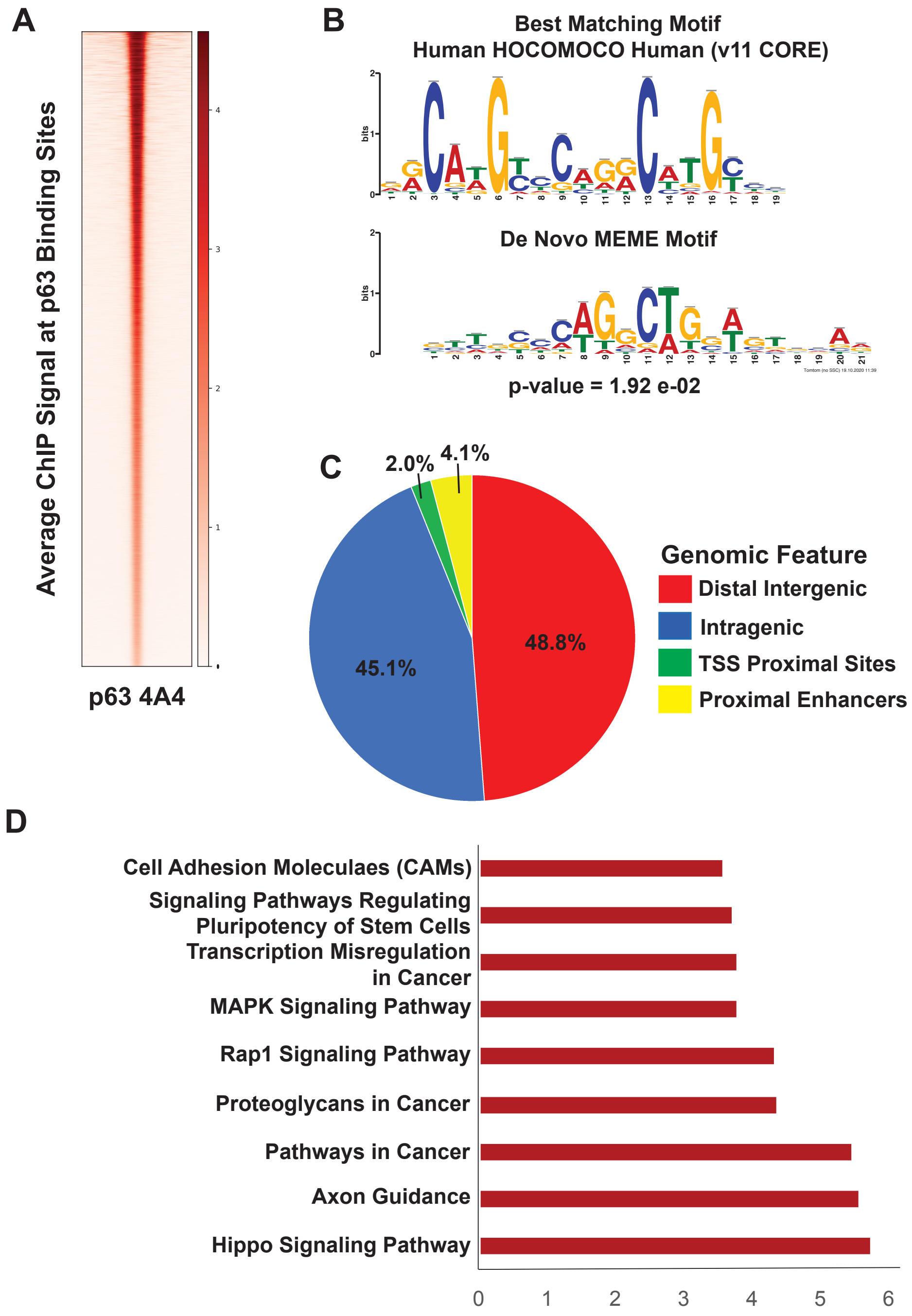

Supplementary Figure 3: ChIP-seq analysis reveals direct p63 targets in SCC152 cells. **(A)** Heatmap of ChIP-seq signal of p63 binding sites. **(B)** The top motif derived from motif analysis on SCC152 p63 ChIP peaks. **(C)** Distribution pattern of p63 binding sites across the genome. **(D)** Bargraphs displaying selected top enriched KEGG pathways associated with genes identified through GREAT analysis of the Top 2500 p63 ChIP-seq peaks.

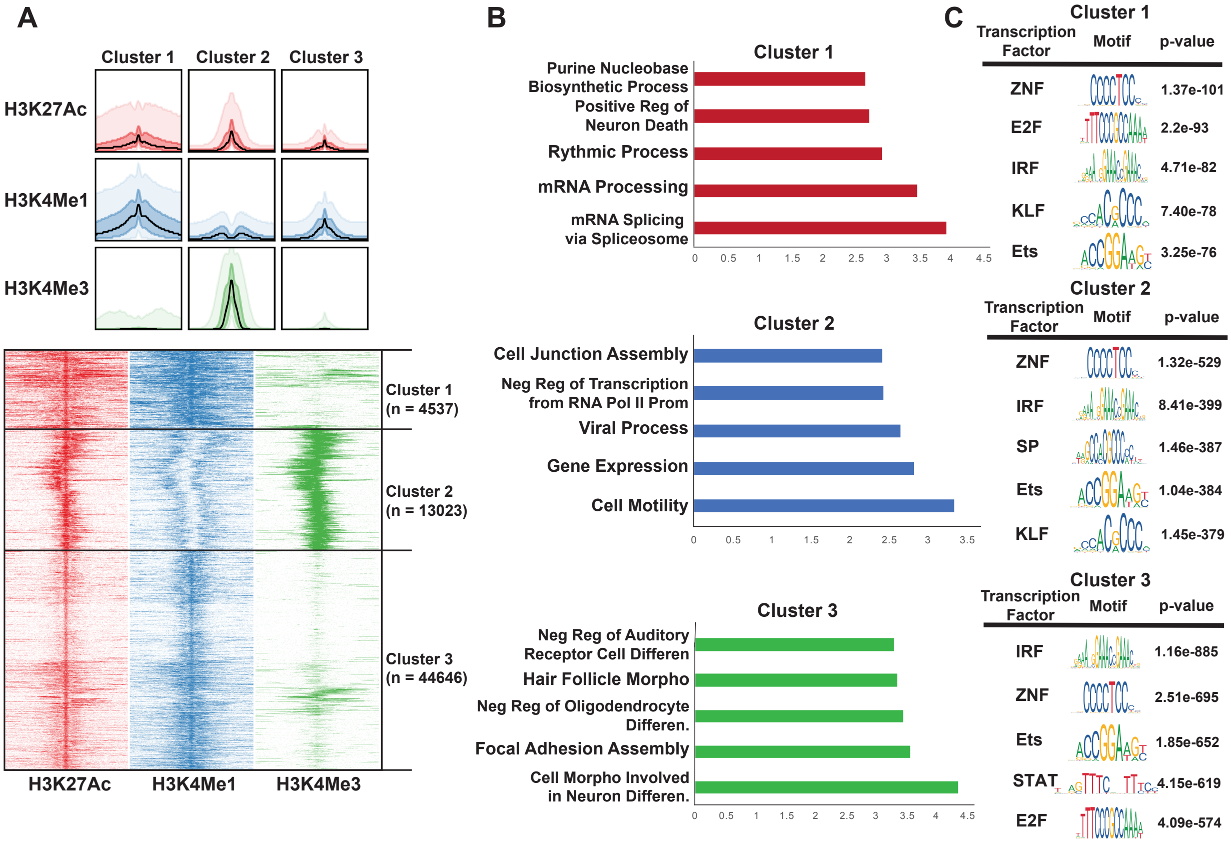

Supplementary Figure 4: K-means clustering of epigenomic landscape in SCC104. (A) Bandplot and heatmap of histone modification signal density using k-means clustering on histone marks in SCC104 showing three different groups. **(B)** Bargraphs displaying top enriched GO biological processes associated with genes annotated to 3 clusters of epigenomic marks. **(C)** The top motifs derived from SCC104 H3K27ac peaks in each identified cluster by AME motif analysis.

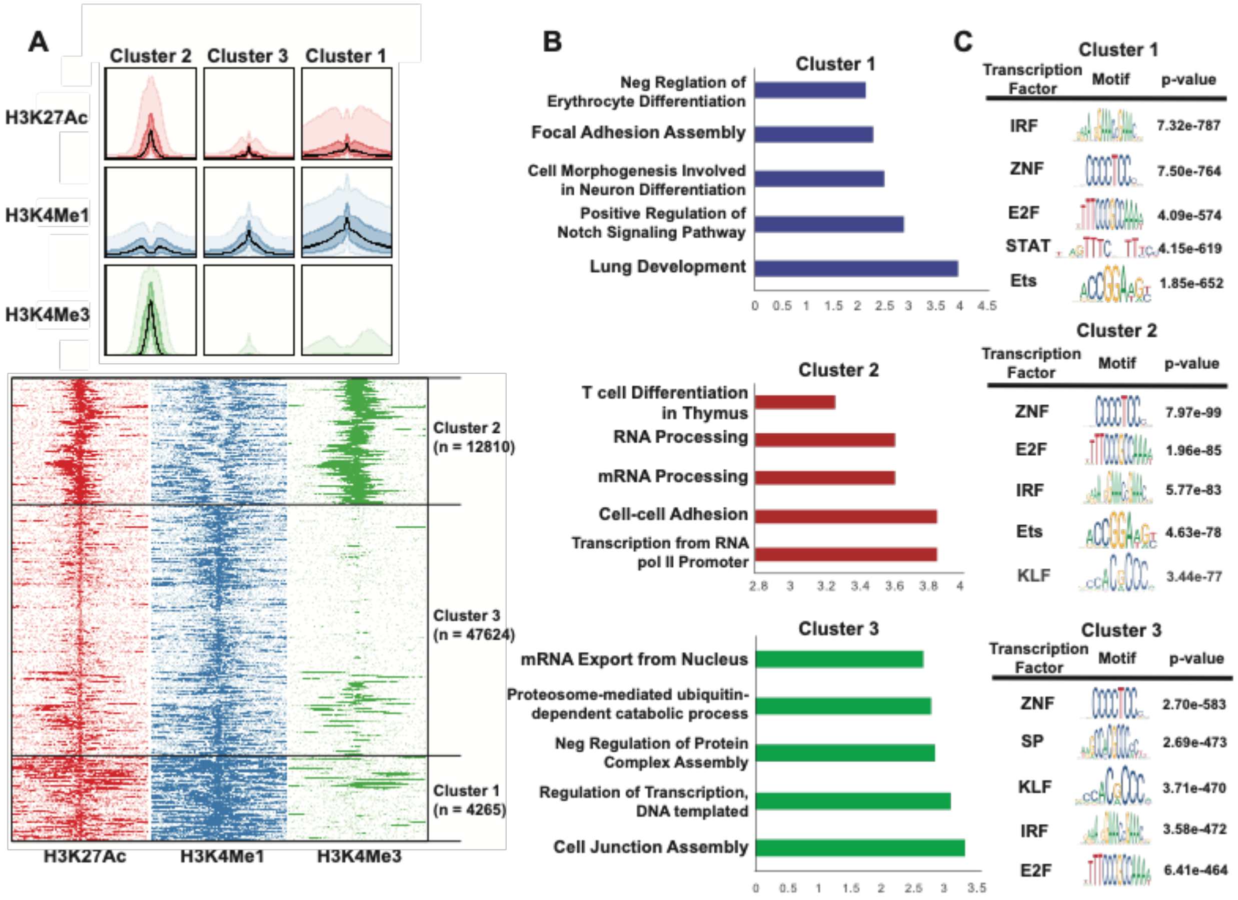

Supplementary Figure 5: K-means clustering of epigenomic landscape in SCC152. (A) Bandplot and heatmap of histone modification signal density using k-means clustering on histone marks in SCC152 showing three different groups. **(B)** Bargraphs displaying top enriched GO biological processes associated with genes annotated to 3 clusters of epigenomic marks. **(C)** The top motifs derived from SCC152 H3K27ac peaks in each identified cluster.

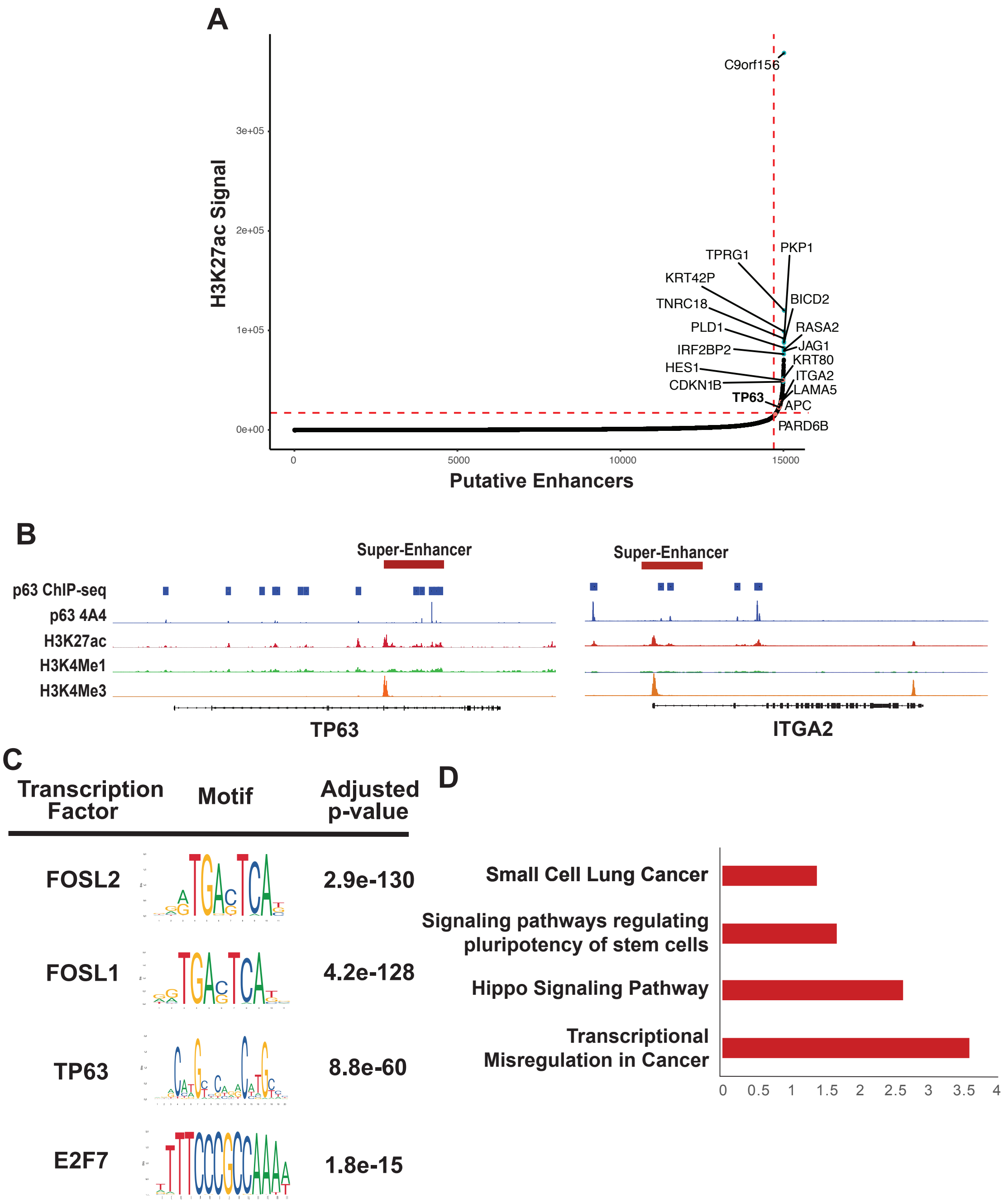

Supplementary Figure 6: p63 binding enriched at super-enhancers in SCC152. (A) Hockey plot displaying the ranked H3K27ac ChIP-seq signal in SCC152. *TP63* is bolded as a super-enhancer associated gene in SCC152. Other labeled points are genes that have been found in previous literature to be associated with HPV infection. **(B)** Top enriched transcription factor motifs found in super-enhancer regions of SCC152. **(C)** Bargraphs displaying selected top enriched KEGG pathways associated with genes identified through ROSE analysis of the super-enhancer landscape in SCC152. **(D)** Histone ChIP data and p63 ChIP-seq data from SCC104 around the *TP63* locus.

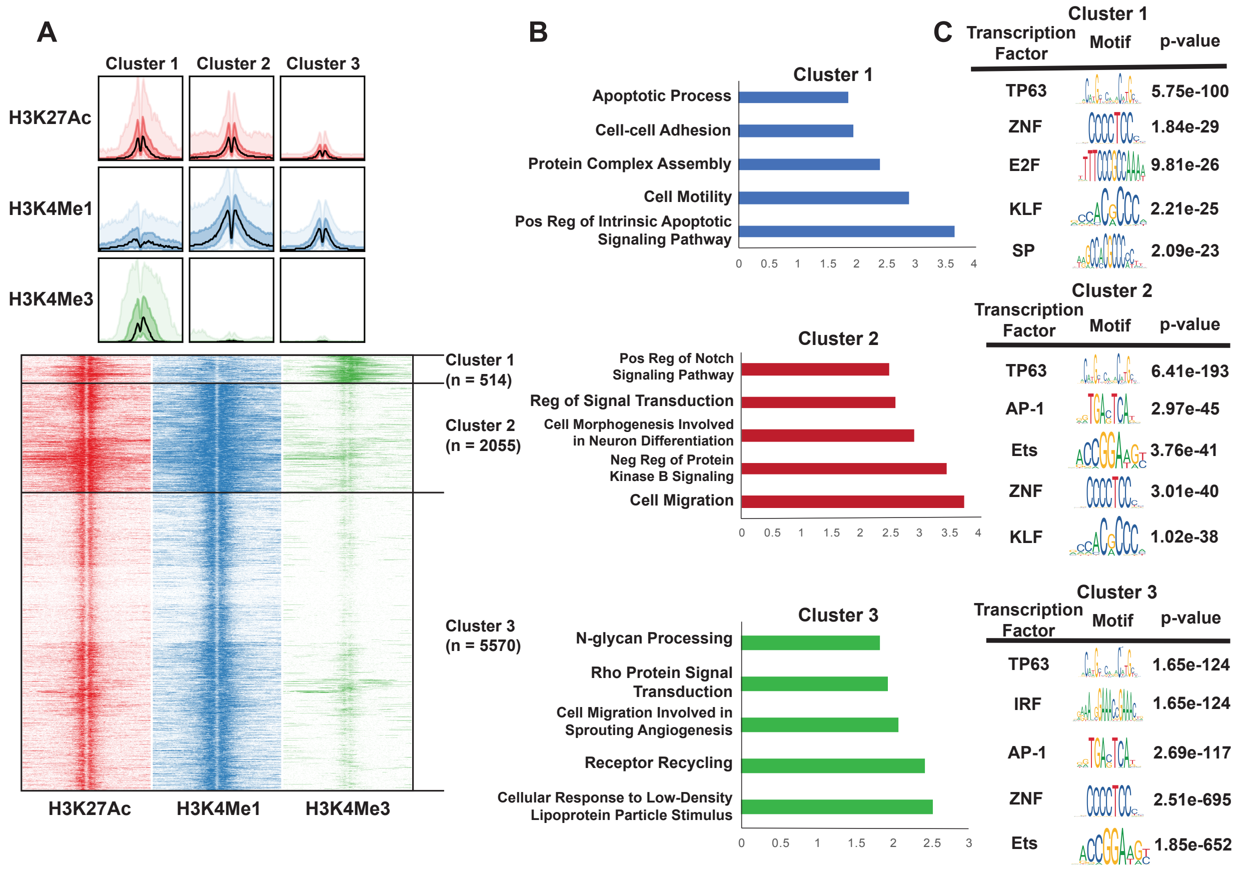

Supplementary Figure 7: K-means clustering of enhancer marks around p63 binding sites. **(A)** Bandplot and heatmap of histone modification signal density using k-means clustering on SCC104 p63 binding sites showing three different groups. **(B)** Bargraphs displaying top enriched GO biological processes associated with genes annotated to 3 clusters of p63 ChIP-seq peaks. **(C)** The top motifs derived from SCC104 p63 ChIP-seq peaks in each identified cluster by AME motif analysis.

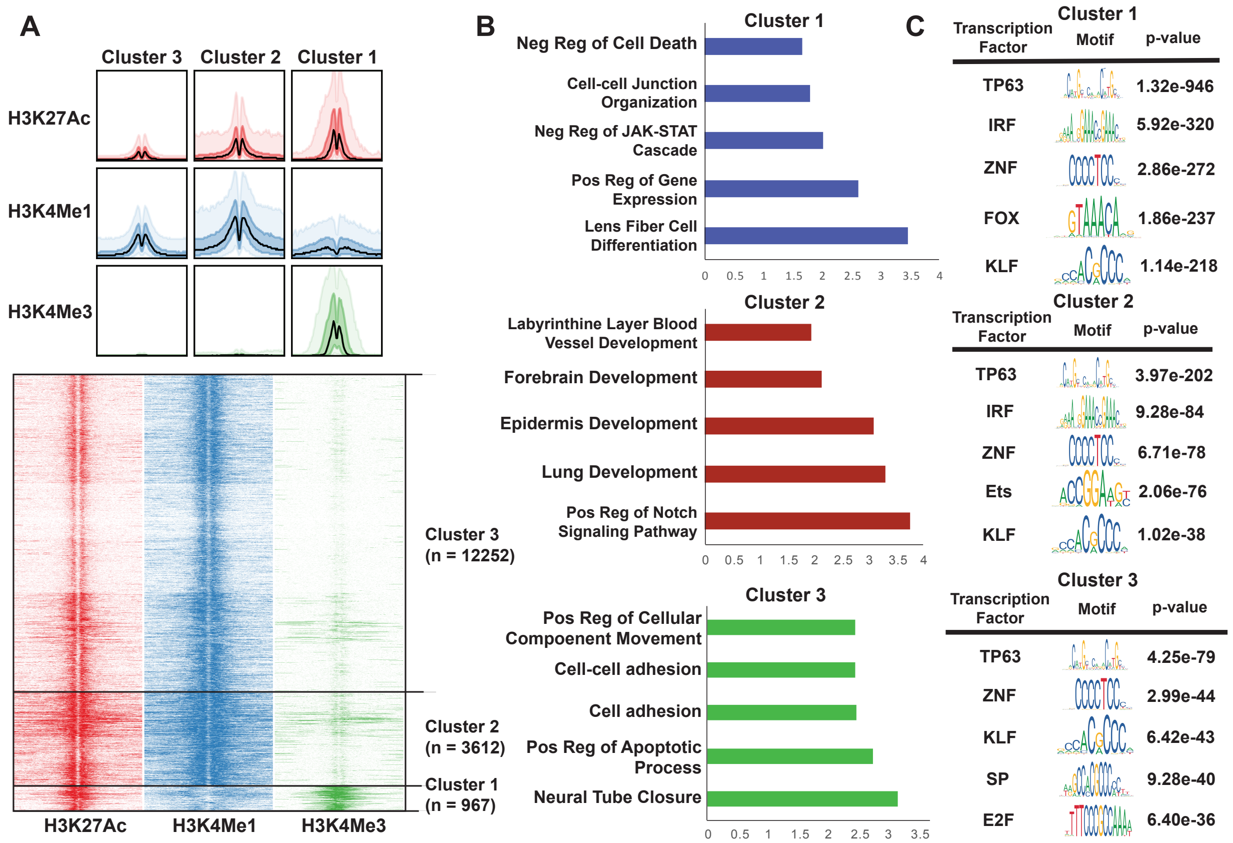

Supplementary Figure 8: K-means clustering of enhancer marks around p63 binding sites. **(A)** Bandplot and heatmap of histone modification signal density using k-means clustering on SCC152 p63 binding sites showing three different groups. **(B)** Bargraphs displaying top enriched GO biological processes associated with genes annotated to 3 clusters of p63 ChIP-seq peaks. **(C)** The top motifs derived from SCC152 p63 ChIP-seq peaks in each identified cluster.

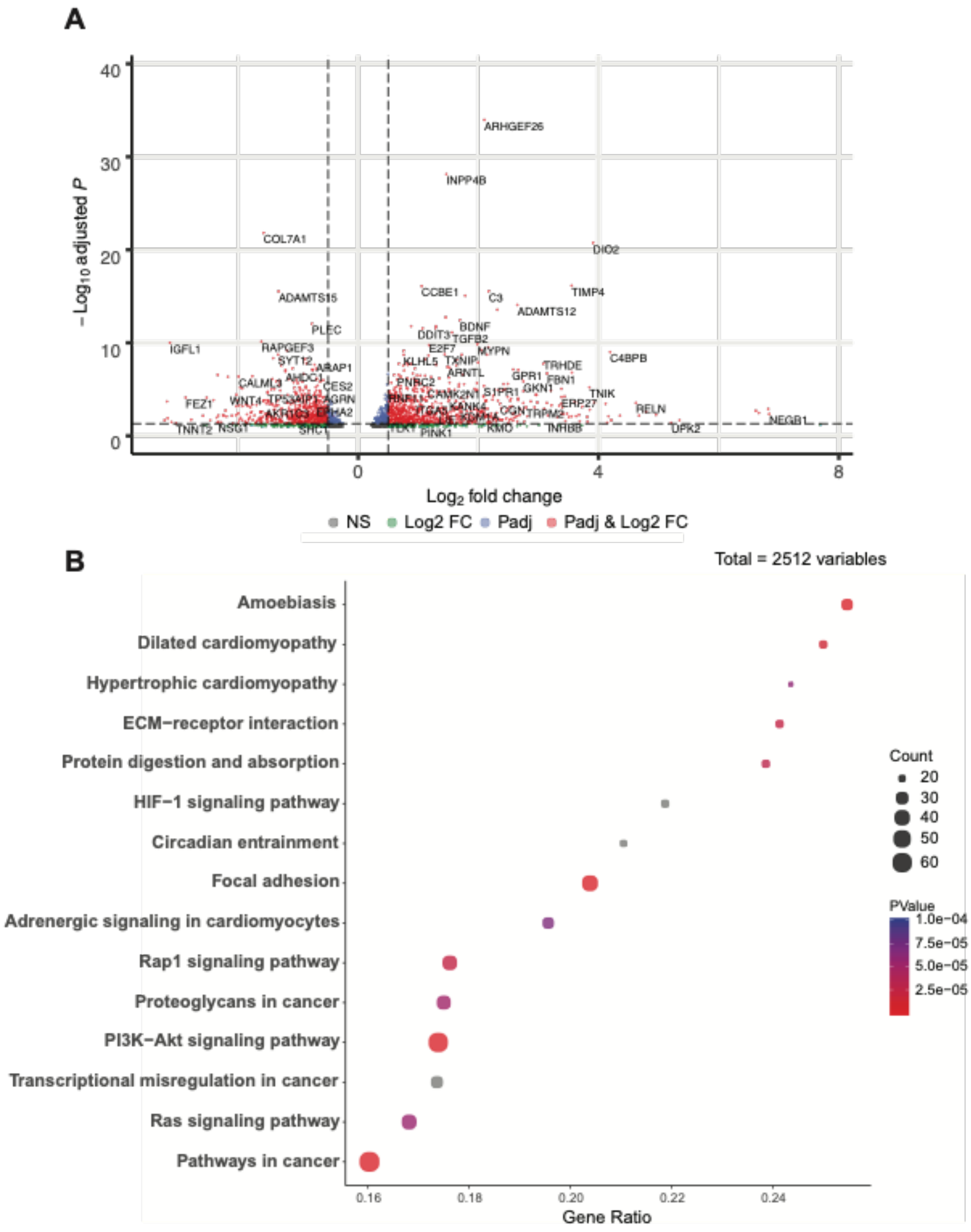

Supplementary Figure 9: p63 knockdown in SCC152 shows enrichment of HPV-associated signaling pathways. (A) Volcano plot of SCC152 p63 knockdown differentially expressed genes. **(B)** KEGG Pathway analysis of SCC152 differentially expressed genes whose expression changed at least by a log2 fold change of one.

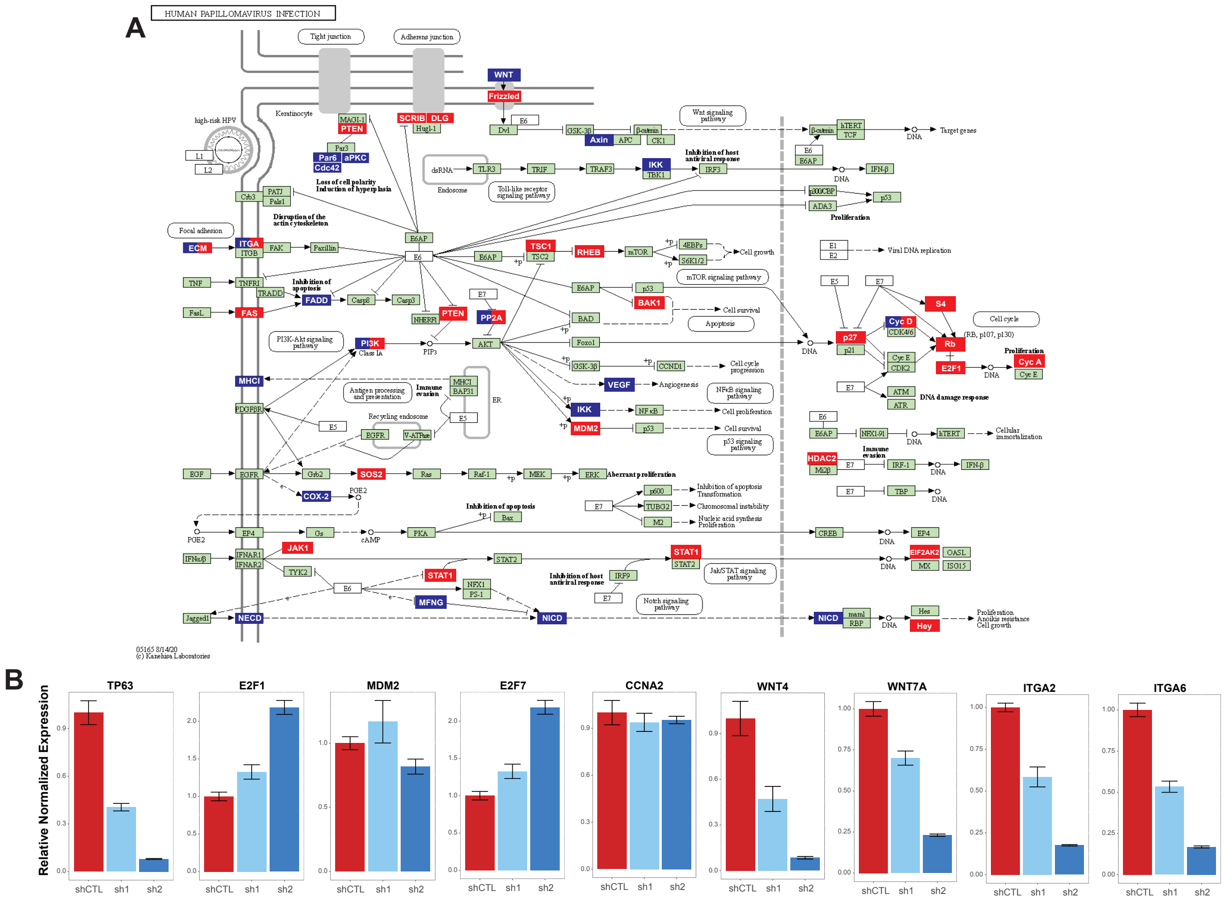

Supplementary Figure 10: p63 regulates the expression of several genes involved in the human HPV infection pathway. (A) Map of established HPV infection pathway players adapted from the KEGG Human Papillomavirus Infection map. Genes and pathways that show upregulation upon loss of p63 as seen in our p63 knockdown RNA-sequencing data are highlighted in red, while genes and pathways that show downregulation upon loss of p63 are highlighted in blue. **(B)** qPCR expression analysis of SCC104 shCTL and shTP63 cell lines. Graphs show the relative normalized expression of genes associated with the HPV infection pathway.

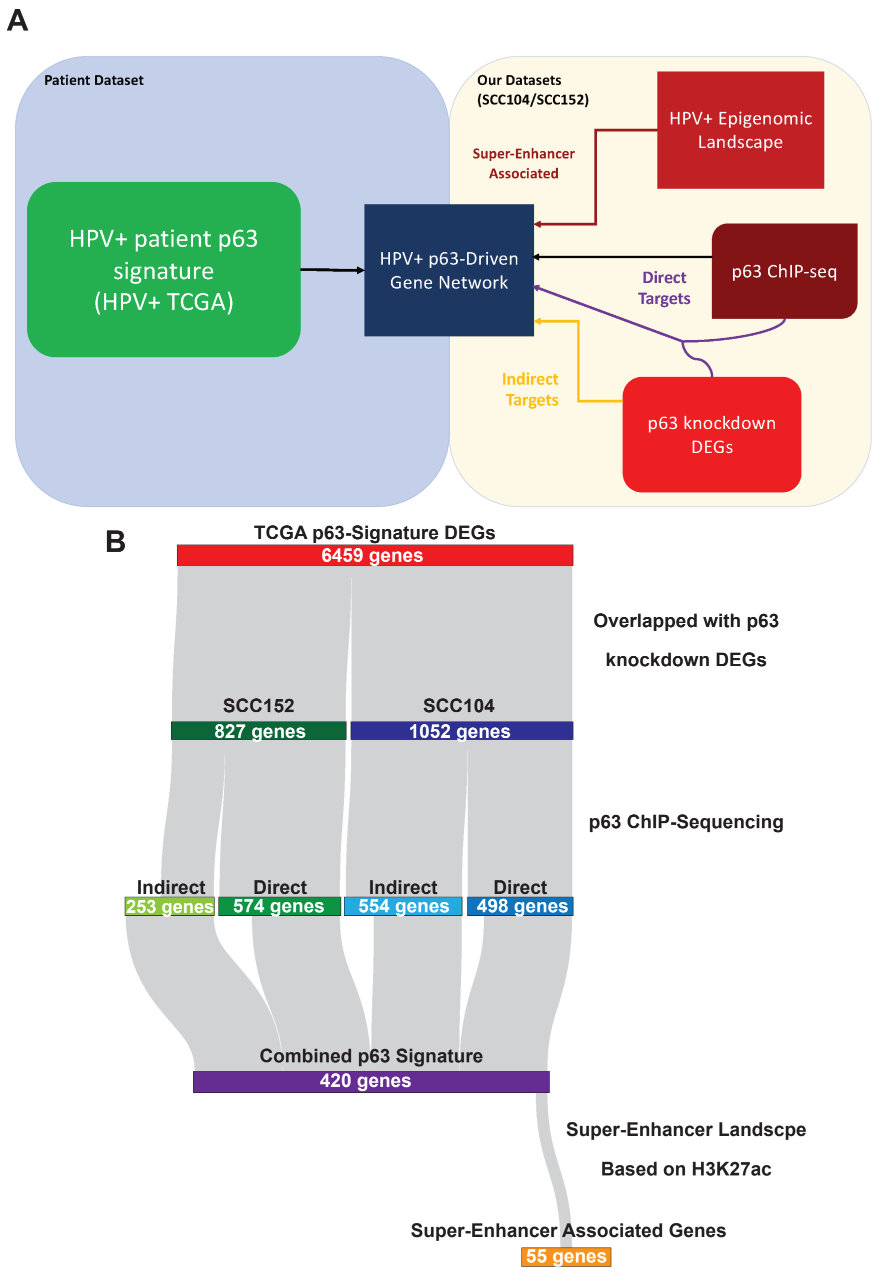

Supplementary Figure 11: Generating a p63-driven gene signature from patient and cell line datasets. **(A)** Flow-chart of undertaken data analysis. The publicly available TCGA HNSCC RNA-sequencing datasets of patient tumor samples as well as our cell line-based data was used to generate DEGs based on p63 expression. This data was then incorporated with our cell line-based datasets to identify genes which are both HPV-specific and relevant to p63 biology. (B) Sankey plot of the generation of the combined p63 signature.

A HPV Pathway

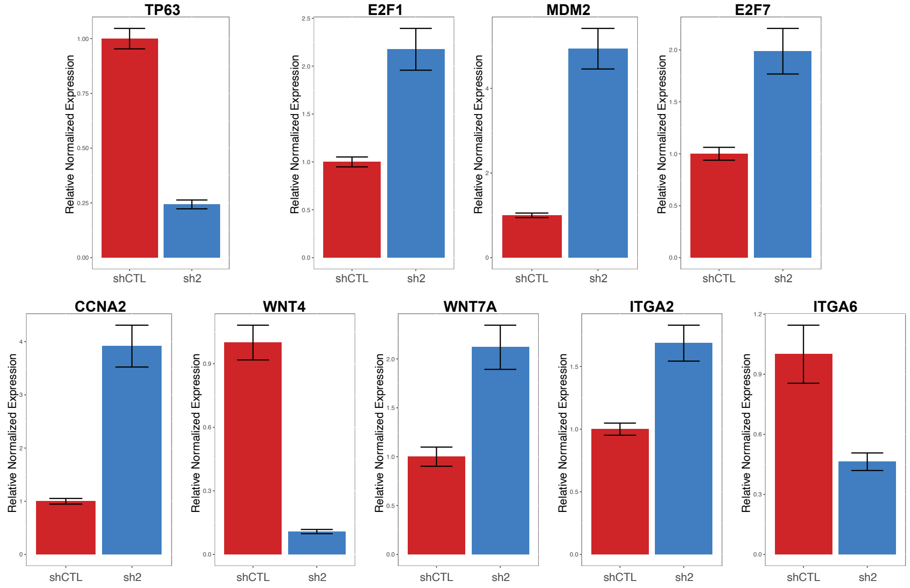

B PI3K Signaling

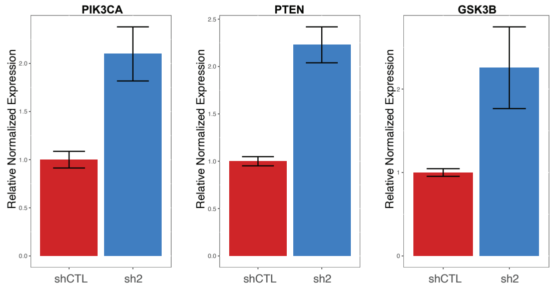

C Subtype Specific Gene Signatures

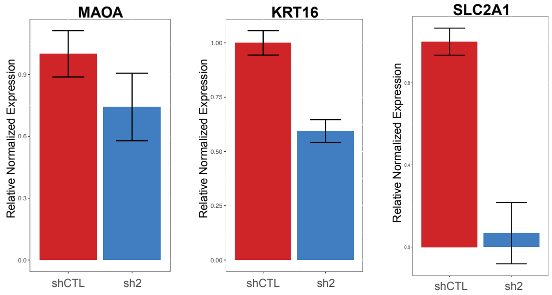

Supplementary Figure 12: (A) qPCR of SCC152 p63 knockdown for genes involved in the HPV infection pathway. **(B)** qPCR of SCC152 p63 knockdown for genes involved in the PI3K signaling pathway. **(C)** qPCR of SCC152 p63 knockdown for genes found in the Zhang et al. and Keck et al. subtype specific gene expression signatures.
